# NY-ESO-1 facilitates anoikis resistance and tumor metastasis by hijacking deubiquitinase OTUB1 to stabilize PP1α

**DOI:** 10.1038/s41419-025-08017-w

**Published:** 2025-10-06

**Authors:** Pengchao Zhang, Jian Cheng, Zhao Liu, Funmilayo O. Adeshakin, Liujiang Dai, Xiangyun Niu, Ziyang Zhang, Xixia Peng, Long Li, Maoxuan Liu, Dehong Yan, Xiaolu Yang, Xiaochun Wan, Guizhong Zhang

**Affiliations:** 1 Center for Protein and Cell-based Drugs, Institute of Biomedicine and Biotechnology, Shenzhen Institutes of Advanced Technology, Chinese Academy of Sciences, Shenzhen, People’s Republic of China; 2 University of Chinese Academy of Sciences, Beijing, PR China; 3 Department of Cancer Biology and Abramson Family Cancer Research Institute, Perelman School of Medicine, University of Pennsylvania, Philadelphia, PA USA

**Keywords:** Ubiquitins, Intracellular signalling peptides and proteins, Oncogene proteins, Oncogenes, Apoptosis

## Abstract

Anoikis resistance, an essential prerequisite for tumor metastasis, is now recognized as a promising target in the fight against tumor progression. However, the detailed mechanisms of anoikis resistance are not fully understood, and drugs targeting anoikis resistance are not currently available. Here we report that NY-ESO-1, a well-known cancer-testis antigen, is linked to a poor prognosis in tumor patients and that it is crucial for anoikis resistance and tumor metastasis. Overexpression of NY-ESO-1 in cancer cells enhanced ERK1/2 activation, which in turn promoted resistance to anoikis, increased colony formation in soft agar, and facilitated lung metastasis in mice. Conversely, NY-ESO-1 knockdown significantly reduced ERK1/2 activity, leading to enhanced anoikis, diminished colony formation, and impaired metastatic potential. Mechanistically, NY-ESO-1 acts as a scaffold protein to recruit the deubiquitinase OTUB1 to PP1α, forming a ternary complex that prevents PP1α from being ubiquitinated. The OTUB1’s deubiquitinase activity, not its ability to suppress E2 enzymes, is necessary for reducing polyubiquitination and improving PP1α stability. Finally, accumulated PP1α proteins significantly activate downstream ERK1/2. Blockade of ERK1/2 or knocking down PP1α antagonized NY-ESO-1-mediated anoikis resistance. These results not only reveal a previously unrecognized mode for deubiquitinase substrate expansion but also highlight the function of NY-ESO-1 in anoikis resistance and suggest NY-ESO-1 as a novel attractive target for preventing tumor metastasis.

## Introduction

Cancer is the most common cause of death worldwide [1], and the majority of cancer deaths are due to metastasis [2]. Metastasis is a multistep process including the dissociation of cancer cells from primary sites, their traversal and survival in the circulatory system, and their proliferation in distant target organs [3]. While all of these steps are required for the development of metastasis, the ability of tumor cells to survive in the vascular system is regarded as the most critical step [4, 5]. In normal cells, loss of contact with the extracellular matrix or neighboring cells leads to an apoptotic process known as anoikis [6, 7]. However, cancer cells typically acquire anoikis resistance, enabling them to survive after detachment from the primary sites, traverse the circulatory and lymphatic systems, and disseminate throughout the body [3, 8]. Therefore, elucidation of the mechanisms and key regulators required for anoikis resistance has important implications for preventing and managing tumor metastasis.

New York esophageal squamous cell carcinoma 1 (NY-ESO-1) is a cancer-testis antigen (CTA) that is normally expressed in germ and placental cells but re-expressed in various cancers, such as melanoma, ovarian cancer, cervical cancer, and triple-negative breast cancer [9–11]. Due to its relatively restricted expression pattern and potent immunogenicity, NY-ESO-1 has been extensively investigated as a potential target for cancer immunotherapy [10, 12]. Several NY-ESO-1-targeted immunotherapies have shown promising anti-tumor effects in pre-clinical and clinical trials [10, 12]. However, even if approved, these therapies are likely to benefit only a small subset of patients, as their efficacy is often limited in advanced cancer patients with poor systemic immune status. Therefore, directly targeting the biological function of NY-ESO-1 to control tumor progression represents an appealing alternative or complementary therapeutic approach. This strategy is currently limited by our poor understanding of NY-ESO-1’s biological role. Despite its association with metastasis [13–15], it remains unclear whether NY-ESO-1 functionally drives metastasis, and if so, by what specific molecular mechanisms.

In this study, we investigate the role of NY-ESO-1 in anoikis resistance and tumor metastasis. We find that NY-ESO-1 strongly enhances ERK1/2 activation, thereby suppressing anoikis and promoting soft agar colony formation in diverse tumor cell lines. The effect of NY-ESO-1 is mediated by PP1α, a serine/threonine-specific protein phosphatase known to be involved in the regulation of MAPK activation [16]. NY-ESO-1 interacts with PP1α and reduces its K48-polyubiquitin modification and subsequent degradation by engaging the deubiquitinase OTUB1, leading to PP1α accumulation and ERK1/2 activation. Consequently, NY-ESO-1 knockdown significantly promoted anoikis and impaired tumor metastasis. Importantly, NY-ESO-1 expression is linked to a poor prognosis in tumor patients, particularly in those with immune-cold tumors, reflecting the clinical significance of NY-ESO-1-associated metastasis. These results establish a critical role for NY-ESO-1 in anoikis resistance and suggest NY-ESO-1 as an attractive target for preventing tumor metastasis.

## Materials and Methods

### Reagents

DMEM medium, L-glutamine, trypsin-EDTA, and fetal bovine serum (FBS) were purchased from Hyclone (Logan, USA); propidium iodide (PI) and 7-AAD from Biolegend (San Diego, CA, USA); PD98059 from Selleck (Houston, TX, USA); trametinib (HY-10999) from MedChemExpress; polybrene (hexadimethrine bromide), chloroquine (CQ), and polyHEMA (Poly(2-hydroxyethyl methacrylate) from Sigma (St. Louis, MO, USA); cycloheximide (CHX) from Aladdin (Shanghai, China); western stripping buffer, MG-132, and crystal violet from Beyotime (Shanghai, China).

### Bioinformatics analysis

The overall survival probability of patients with high and low NY-ESO-1 or PP1α expression was analyzed using the online Kaplan-Meier Plotter (https://kmplot.com/analysis/) and calculated by log-rank test. Cohorts of patients were split by median expression values through auto-select best cut-off. Gene chip mRNA data from breast cancer (systemically untreated patients) [17], lung cancer [18], gastric cancer [19], myeloma [20], ovarian cancer [21], and colon cancer [20], as well as proteomic data from Breast cancer (Tang 2018 cohort) [22] were used in this study.

### Cell culture

HeLa, MDA-MB-231, MCF-7, A375, A549, and HEK293T cells were obtained from the Cell Bank of the Chinese Academy of Sciences (Shanghai, China) or ATCC (Manassas, Virginia, USA) and were authenticated by the vendor using short tandem repeat (STR) profiling. NCI-H1299 and SW620 were obtained from ServiceBio (Wuhan, China). All cell lines cultured in DMEM or 1640 medium supplemented with 10% FBS and 2 mmol/L L-glutamine, and checked for the absence of Mycoplasma using PCR method.

### Plasmids and siRNAs

NY-ESO-1 (CTAG1B, NM_001327) was transiently expressed using pLVX-based plasmids or stably expressed using pLVX-based lentiviral vectors. The full-length human NY-ESO-1 cDNA was generated from A375 cells by RT-PCR, digested with EcoRⅠ and BamHI, and cloned into the pLVX vector. PP1α-flag expression plasmid (pENTER-PPP1CA-Flag-His) was purchased from WZ Biosciences Inc. (Shandong, China). OTUB1-HA expression plasmid (pCDNA3.1-OTUB1-HA) was purchased from Hanyi Biological Technology Co., LTD (Guangzhou, China). Truncations of NY-ESO-1, PP1α and OTUB1 were constructed by the PCR-based method, and their sequences were verified. The plasmids encoding PP1α mutants, in which the three ubiquitination sites (Lys-6, Lys-24 and Lys-305) were replaced either individually or completely by Arg, and the plasmids encoding OTUB1 mutants, in which Asp-88 and Cys-91 were replaced by Ala and Ser, respectively, were generated by PCR-based site-directed mutagenesis. Control siRNA (siCT), siRNAs targeting human PP1α (siP-1 and siP-2), OTUB1 (siOT-1 and siOT-2), and USPs (siUSP11-1, siUSP11-2, siUSP47, siUSP9X, siUSP10, siUSP5), and shRNAs targeting human NY-ESO-1 (shNY-1 and shNY-2) were synthesized by GenePharma (Suzhou, China). To construct the lentiviral NY-ESO-1 shRNA expression vector, synthesized oligo pairs were annealed and subcloned into the pLL3.7 vector. The sequences of shRNAs targeting NY-ESO-1 were 5’- GCTTCAGGGCTGAATGGAT-3’ (shNY-1) and 5’-CCGGCAACATACTGACTAT-3’ (shNY-2). The sequences of siRNAs are provided in Table S1.

### Transfection and lentiviral packaging

Transfection was performed using Lipofectamine 3000 according to the manufacturer’s protocol (Invitrogen). The lentiviruses were packaged by co-transfecting each lentiviral vector together with helper plasmids Gag, Rev, and VSVG into HEK293T cells. 72 h after transfection, virus-containing medium was collected by centrifugation at 100 × g for 5 min and concentrated by centrifugation at 50,000 × g for 2 h.

### Generation of stable cell lines

To generate NY-ESO-1 or specific shRNA-expressing stable cell lines, HeLa, MCF-7, MDA-MB-231, SW620, NCI-H1299, A549, and A375 cells were transduced using pLVX-based or pLL3.7-based lentiviruses with 6 mg/ml polybrene and selected with FACS. The pLL3.7 and pLVX vectors were used to create the corresponding control stable cells.

### Anoikis induction

Cells (0.8–1.0 × 10^5^ for a 12-well plate or 5 × 10^5^ for a 6-well plate) were plated in poly-HEMA-coated plates and allowed to grow for the indicated durations. Cells were collected for FACS detection (using FACS Canto II, BD Bioscience or CytoFLEX, Beckman) after being trypsinized into single cells and labeled with PI or 7-AAD (12 min, 4 °C in the dark), or collected for protein extraction and subsequent immunoblotting.

### Soft agar colony formation assay

Briefly, 2 mL 0.5% low melting point agar (Sangon Biotech, Shanghai, China) solution in complete culture medium was plated in 6-well plates for each well and allowed to solidify at room temperature for 30 min. Then overlaid with 2 mL 0.3% agar solution in complete culture medium containing 1 × 10^4^ cells. Plates were allowed to solidify at room temperature and then kept in a 37 °C humidified cell culture incubator. To prevent desiccation, 200 μL of medium was added to each well twice weekly. After 2–4 weeks, colonies were photographed under a microscope (Olympus, Tokyo, Japan) and visualized by staining with 0.005% crystal violet (Beyotime) for 1 h. The number of colonies was counted using ImageJ 1.51j8 software (NIH, USA).

### Protein extraction and immunoblotting

Whole-cell lysates were prepared by suspending cells in RIPA buffer (Beyotime) supplemented with 1× complete protease inhibitors mixture and 1× phosphatase inhibitor (Roche, Basel, Switzerland). Protein concentration was determined by BCA assay (Pierce, Massachusetts, USA). Equal quantities of proteins were separated by SDS-PAGE, transferred to a PVDF membrane, and blotted with specific antibodies. Proteins in the membrane were visualized by an enhanced Chemiluminescence Detection Kit (Millipore, Massachusetts, USA). Rabbit antibodies against the following proteins or modifications were used with the catalog numbers and sources indicated: NY-ESO-1 (GTX100503, GeneTex, Irvine, CA); ERK1/2 (4695, CST, Beverly, MA); Phospho-ERK1/2 (4370, CST); Phospho-MEK1/2 (9121, CST); Phospho-B-Raf (2696, CST); Phospho-C-Raf (9427, CST); DUSP2 (27327-1-AP, Proteintech); MEK1/2 (9122, CST); K48-linkage specific polyubiquitin antibody (ab190061, Abcam, Cambridge, MA); OTUB1 (YT5679, Immunoway); Mouse antibodies against the following proteins/epitopes were used: NY-ESO-1 (sc-53869, Santa Cruz, Dallas, TX); PP1α (sc-7482, Santa Cruz); DUSP4 (66349-1-Ig, Proteintech, Wuhan, China); GAPDH (MB001; Bioworld Technology, Bloomington, MN); β-actin (A5441; Sigma); β-Tubulin (T200608, Zenbio, Chengdu, China); HRP-mouse anti-Myc (SMC-255D-HRP) from Stressmarq (Victoria, BC, Canada); HRP-conjugated mouse anti-Flag (200-303-383) from Rockland (Limerick, PA); HRP-conjugated goat anti-mouse IgG (074-1806) from KPL (Taiwan, China), and HRP-conjugated goat anti-rabbit IgG (E030120-02) from EARTHOX (Millbrae, CA). Uncropped Western blots are provided in the supplemental file titled “original data”.

### Immunoprecipitation

A375 or transfected HEK293T cells were lysed in immunoprecipitation (IP) buffer (150 mM NaCl, 50 mM Tris-HCl, pH 7.4, 50 mM EDTA, 1.0% NP-40, 1 mM PMSF) supplemented with 1× complete protease inhibitors mixture (Roche). Immunoprecipitation (IP) assays were performed as described [23] using specific antibodies (Mouse anti-NY-ESO-1, sc-53869, Santa Cruz; or mouse anti-Myc, M4439, Sigma; or mouse anti-Flag, F3165, Sigma; or mouse anti-HA, H3663, Sigma) or Ig control, and detected by immunoblotting.

### LC-MS/MS protein identification

HEK293T cells were transfected with PP1α-Flag, Myc-NY-ESO-1 alone or in combination and harvested 48 h later. Cell lysates were immunoprecipitated with anti-Myc or anti-Flag antibodies, and the immunoprecipitates were identified using LC-MS/MS by Luming Bio (Shanghai, China). The complete lists of proteins identified are shown in Table S2.

### RNA-Seq

Total RNA extracted from MCF-7 cells stably transfected with NY-ESO-1 or control plasmids was used for mRNA library preparation. Completed libraries were sequenced on an Illumina HiSeq instrument by GENEWIZ, Inc. (Suzhou, China). To mine the altered pathways, Gene Set Enrichment Analysis (GSEA) was performed on RNA sequencing data by using the KEGG and REACTOME gene sets obtained from the Molecular Signature Database. The normalized enrichment score (NES) was calculated and used to evaluate the effectiveness of each enrichment in identifying candidate gene sets that influence cellular processes. The complete lists of enriched gene sets are presented in Table S3.

The raw sequence data reported in this paper have been deposited in the Genome Sequence Archive [24] in National Genomics Data Center [25], China National Center for Bioinformation / Beijing Institute of Genomics, Chinese Academy of Sciences (GSA-Human: HRA011765) that are publicly accessible at https://ngdc.cncb.ac.cn/gsa-human.

### Ubiquitination assay

Cells were treated with MG-132 for 4–6 h and lysed in 1% SDS (50 mM Tris-HCl, pH 7.4, 50 mM DTT) supplemented with 1× complete protease inhibitors mixture (Roche) and 10 mM N-ethylmaleimide (NEM, Sigma). After boiling for 5 min, lysates were diluted 10 times with cold IP buffer supplemented with 1× protein inhibitor and 10 mM NEM, and subject to immunoprecipitation with PP1α antibody or Flag antibody. The immunoprecipitates were resolved by 8% SDS-PAGE and transferred to the PVDF membrane. After blocked in 5% BSA, the blot was probed with a K48-linkage specific polyubiquitin antibody (ab190061, Abcam).

### Cycloheximide chase assay

Cells were treated with 50 µg/mL cycloheximide (CHX) and then incubated at 37 °C for the indicated time points. Cell pellets were collected for protein degradation analysis using immunoblotting. The results were quantified by densitometry using Image J software.

### Experimental lung metastasis

NCG mice at 6-8 weeks of age were purchased from GemPharmatech (Nanjing, China) and housed in the Shenzhen Institutes of Advanced Technology animal facility under pathogen-free conditions. Mice were intravenously injected with human melanoma cells (A375) stably expressing NY-ESO-1-specific shRNAs (shNY-1) or control shRNAs (shCT), respectively (1.5×10^6^ cells in 100 μL PBS). After 21 days, the mice were euthanized and the lungs were excised, and photographed. Metastatic colonies on the lung were counted macroscopically under a dissecting microscope.

To study how NY-ESO-1 expression affects the metastasis potential of tumor cells in vivo, 6-8 weeks old female NCG mice were intravenously injected with MDA-MB-231 cells stably expressing NY-ESO-1 or their corresponding control cells (1.0×10^6^ cells in 100 μL PBS). One day after inoculation, mice were treated with either vehicle or trametinib by oral gavage for 5 consecutive days at a dose of 2 mg/kg per day. Mice were euthanized after 24 days, and their lungs were excised and photographed.

### Statistical analysis

All analyses were performed using GraphPad Prism Software. Data were expressed as mean ± SEM. Student’s *t*-test was used to compare continuous data for two groups. The log-rank test was used to examine the differences in patient survival across groups. *P*-values of less than 0.05 were considered to be significant. No blinding was applied for investigators during group allocation, animal use, or outcome assessment throughout the study. Mice were randomly allocated to experimental groups. The sample size was determined empirically based on prior experience with experimental variability, without statistical predetermination.

## Results

### NY-ESO-1 expression is associated with reduced survival in patients with immune-cold tumors

**Fig. 1:**
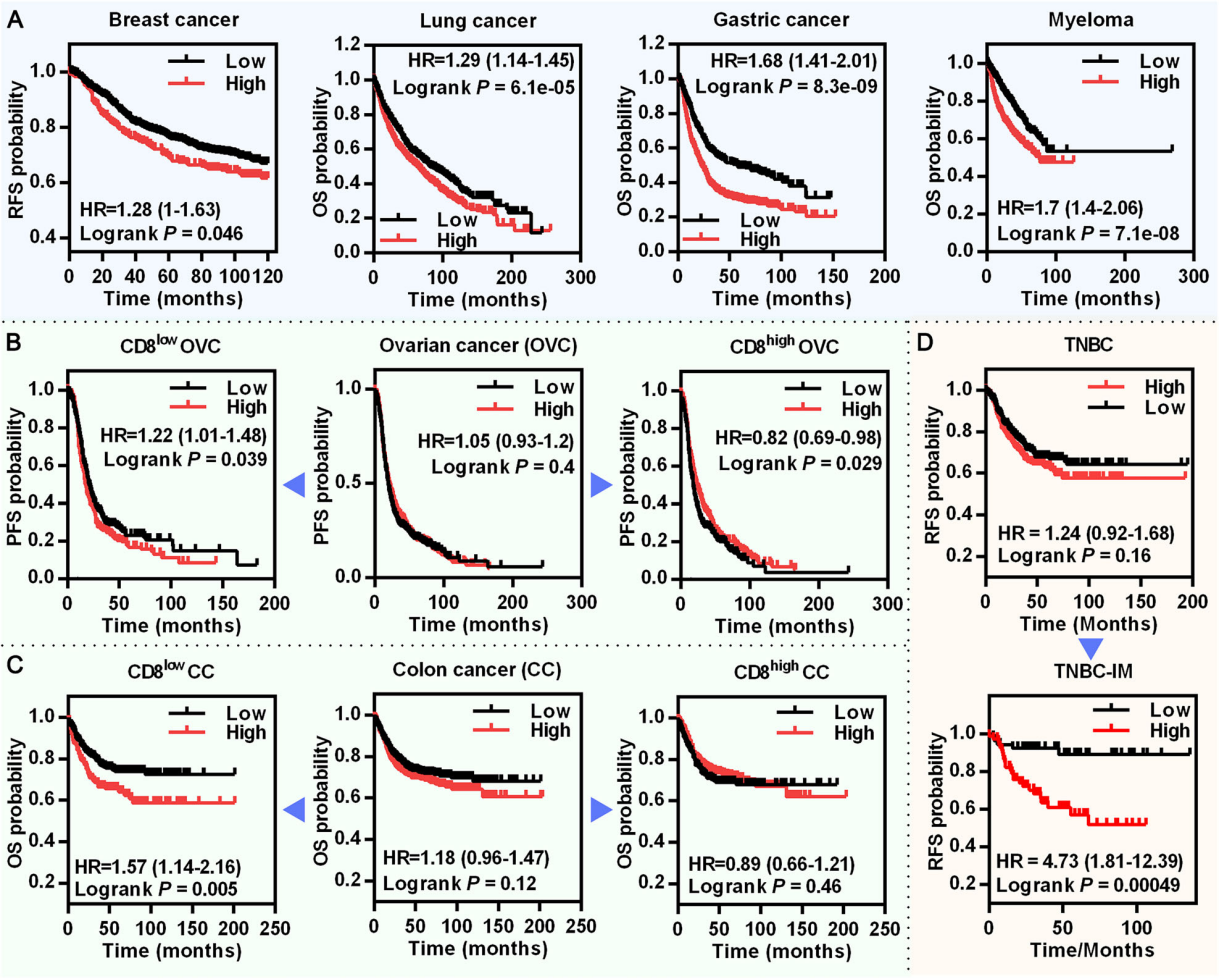
Correlations between NY-ESO-1 expression and survival probability in different cancer cohorts. **A** The survival plots for breast cancer patients (systemically untreated patients, *n* = 1025), lung cancer patients (*n* = 2166), gastric cancer patients (*n* = 875), and myeloma patients (*n* = 1416) with or without NY-ESO-1 (217339_x_at) expression were generated via Kaplan–Meier Plotter, RFS Relapse-free survival, OS Overall survival. **B**, **C** The survival plots for ovarian cancer patients (*n* = 1435) and colon cancer patients (*n* = 1302) with or without NY-ESO-1 (217339_x_at) expression. Correlations between NY-ESO-1 expression and survival probability in the entire cohorts (middle panels) as well as in immune-cold (CD8^low^) and immune-hot (CD8^high^) subgroups (left and right panels, respectively) were shown. CD8^low^ expression value below median, CD8^high^ expression value above median, PFS Progression-free survival. **D** Correlations between NY-ESO-1 expression and survival probability in the entire TNBC cohorts (upper panel) as well as TNBC IM-subtype, IM Immunomodulatory subtype. For (**A**–**C**), the Hazard ratio (HR) and *P*-value for each association are shown within each plot.

Although NY-ESO-1 is a well-known CTA that re-expressed in many cancer types, its value as a prognostic biomarker is in question, and its role in tumor development remains unclear. In some tumors, NY-ESO-1 expression has been linked to a poor clinical prognosis, yet contradictory or no correlations have been found in other tumors [10]. We analyzed the correlations between NY-ESO-1 expression and patients’ survival probability using the Kaplan-Meier Plotter. NY-ESO-1 expression markedly reduced survival for patients with systemically untreated breast cancer, lung cancer, gastric cancer, or myeloma (Fig. 1A), but it had no discernible impact on the survival of patients with ovarian or colon cancer (Fig. 1B, C).

Given that the inherent potent immunogenicity of NY-ESO-1 may influence patient prognosis through immune-related pathways, which could mask its other tumor-promoting functions, we further analyzed the correlation between NY-ESO-1 expression and patient survival in immune-hot and immune-cold subgroups, defined by CD8 expression levels. This stratification is based on the critical role of CD8^+^ T lymphocytes in adaptive anti-tumor immunity, as their infiltration into tumors is commonly linked to tumor regression and favorable clinical outcomes across multiple cancer types. We discovered that high NY-ESO-1 expression markedly decreased the survival rate of ovarian and colon cancer patients with an immune-cold phenotype, but it had no effect on or even improved the survival of immune-hot individuals (Fig. 1B, C). Similarly, high NY-ESO-1 expression markedly increased patient mortality in those with triple-negative breast cancer (TNBC) immunomodulatory (IM) subtype, which is characterized by a predominance of immune pathway dysregulation [26]. By contrast, high NY-ESO-1 expression showed trends to reduce patient survival rates across the entire TNBC cohort, although the differences were not statistically significant (Fig. 1D). These findings imply that NY-ESO-1 may influence both immune and non-immune pathways for tumor progression and is associated with a poor prognosis in patients with immune-cold tumors.

### NY-ESO-1 promotes anoikis resistance, soft agar colony formation and metastasis

More than 90% of patients died from metastasis rather than growth of the primary tumors. NY-ESO-1 expression was strongly associated with tumor metastasis [13–15]. To confirm its metastasis-promoting function, we generated MCF-7, HeLa, and MDA-MB-231 cell lines stably expressing NY-ESO-1. Overexpression of NY-ESO-1 did not exhibit any detectable proliferative effect (Fig. S1A–C). Moreover, NY-ESO-1 slightly inhibited cell migration of MCF-7 and HeLa cells, but it showed a trend to increase migration of MDA-MB-231, although this effect did not reach a statistically significant level (Fig. S1D, E).

**Fig. 2:**
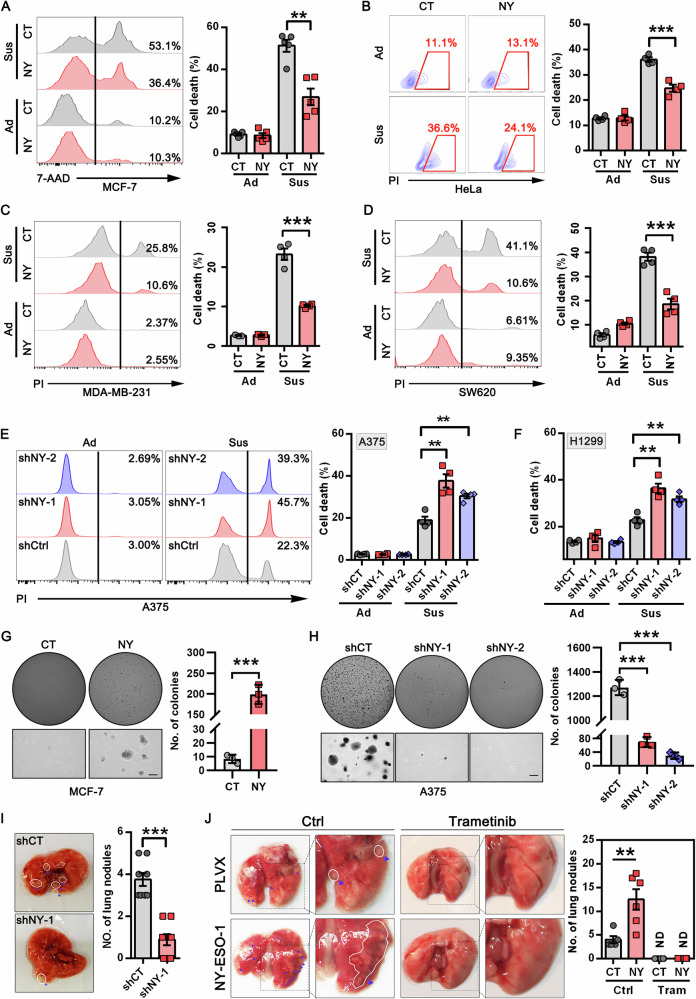
Role of NY-ESO-1 in anoikis and tumor metastasis. **A**–**D** Representative FACS graphs (left) and statistical analysis (right) of cell death in MCF-7 (**A**), HeLa (**B**), MDA-MB-231 (**C**), and SW620 cells (**D**) that were stably transfected with NY-ESO-1 expressing vectors (NY) or control vectors (CT). **E**, **F** Representative FACS graphs (left) and statistical analysis (right) of cell death in A375 (**E**) and H1299 cells (**F**) stably transfected with NY-ESO-1-specific shRNAs (shNY-1 or shNY-2) or control shRNAs (shCT), respectively. For (**A**–**F**), cells were grown under adherent (Ad) or suspension (Sus) conditions for 48–72 h and harvested for FACS-based cell death analysis using 7-AAD or PI staining kit. **P < 0.01; ***P < 0.001. **G** Representative photographs (left) and statistical analysis (right) of colonies formed in soft agar by control and NY-ESO-1-stably expressing MCF-7 cells. Scale bar = 500 μm, ***P <0.001. **H** Representative photographs (left) and statistical analysis (right) of colonies formed in soft agar by control and NY-ESO-1-knockdown A375 cells. Scale bar = 500 μm, ***P < 0.001. **I** Representative photographs (left) and statistical analysis (right) of metastatic nodules present in the lungs from mice inoculated with A375 cells stably transfected with shCT or shNY-1, respectively. Arrow denotes visible metastatic sites. *N* = 6 mice per group, ***P < 0.001. **J** Representative photographs (left) and quantification (right) of metastatic nodules present in the lungs from mice inoculated with indicated MDA-MB-231 cells and treated with vehicle or trametinib (2 mg/kg/d by oral gavage for 5 consecutive days beginning one day after inoculations). The arrows indicate visible metastatic sites. *N* = 5–6 mice per group, **P < 0.005.

Resistance to detachment-induced cell death, or anoikis, which enables tumor cells to survive during circulation, is one of the prerequisites for metastasis [3]. Therefore, we evaluated the role of NY-ESO-1 in anoikis by culturing cells in polyHEMA-coated plates. Of note, NY-ESO-1-expressing MCF-7, HeLa, MDA-MB-231 and SW620 cells showed markedly lower levels of apoptosis compared to their corresponding controls (Fig. 2A–D). Conversely, we knocked down endogenous NY-ESO-1 in A375 and H1299 cells. This resulted in a significant increase in cell death when the cells were cultured in suspension (Fig. 2E, F, and Fig. S1F), although it did not affect adherent proliferation or short-term DNA synthesis in suspension cells (Fig. S1G, H). Moreover, NY-ESO-1-knockdown cells can significantly regain their resistance to suspension cell death upon NY-ESO-1 re-expression (Fig. S1I), further supporting the role of NY-ESO-1 in promoting anoikis resistance.

We next assessed the impact of NY-ESO-1 on anoikis resistance and metastatic potential using a soft agar colony formation assay. Overexpression of NY-ESO-1 significantly enhanced colony formation, increasing both the number and size of colonies (Fig. 2G, and Fig. S2A–C). In contrast, NY-ESO-1 knockdown led to a significant reduction in colony formation (Fig. 2H, and Fig. S2D), further supporting its role in anoikis resistance.

To investigate the role of NY-ESO-1 in tumor metastasis in vivo, we intravenously implanted control and NY-ESO-1-knockdown A375 cells into the severely immunodeficient NCG mice. On day 21 following implantation, NY-ESO-1 knockdown cells generated fewer tumor foci on the lung surface compared with control cells (Fig. 2I), suggesting NY-ESO-1 knockdown suppressed lung metastasis of tumors. Additionally, we used the MDA-MB-231 lung metastasis model to evaluate the effect of NY-ESO-1 expression. The results showed that NY-ESO-1 expression noticeably increases the ability of MDA-MB-231 cells to metastasize to the lung (Fig. 2J). Taken together, these results indicate that NY-ESO-1 plays a crucial role in mediating anoikis resistance and experimental lung metastasis.

### NY-ESO-1 activates ERK1/2 to suppress anoikis

**Fig. 3:**
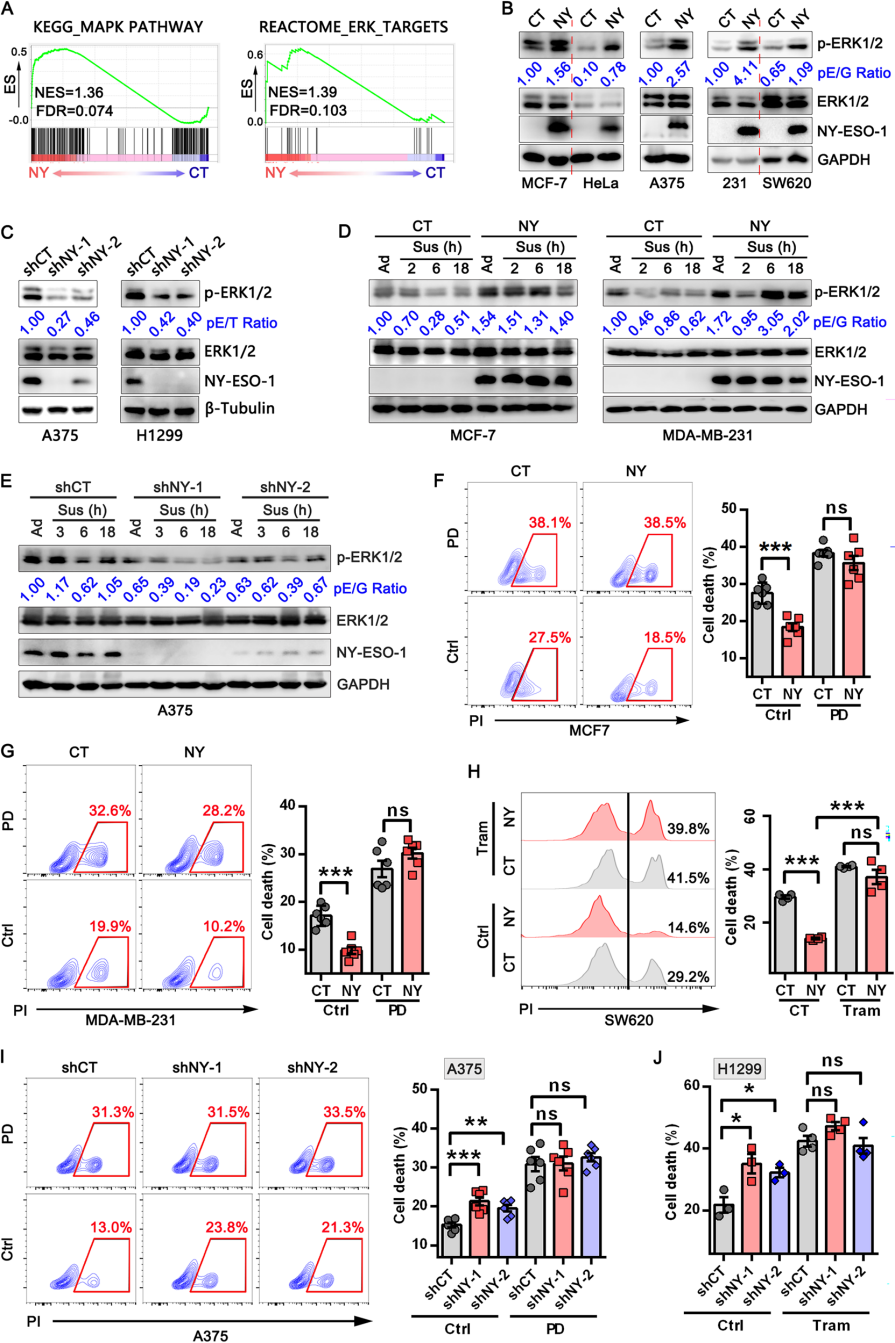
NY-ESO-1 protects cancer cells from anoikis by promoting ERK1/2 activation. **A** GSEA for enrichment of genes involved in MAPK (left) and ERK1/2 pathway (right) in NY-ESO-1 stably transfected MCF-7 cells (NY) versus control cells (CT). **B**, **C** Immunoblot analysis of indicated proteins in tumor cells with or without NY-ESO-1 overexpression or knockdown. Blots of A375 cells in (**B**, **C**) were exposed for different times to better show the effects of NY-ESO-1 overexpression or knockdown. **D** Immunoblot analysis of ERK1/2 activation (p-ERK1/2) in tumor cells stably expressing NY-ESO-1 and control cells grown in adherent (Ad) or suspension (Sus) conditions. **E** Immunoblot analysis of ERK1/2 activation in A375 cells with stable NY-ESO-1 knockdown and control cells cultured under adherent (Ad) or suspension (Sus) conditions. For panels (**B**–**E**), the relative intensity of pERK1/2 normalized to loading controls is shown. The pE/G ratio denotes pERK1/2 normalized to GAPDH, and the pE/T ratio represents pERK1/2 normalized to β-Tubulin. **F**–**H** Representative FACS graphs (left) and statistical analysis (right) of cell death in MCF-7 (F), MDA-MB-231 (**G**), and SW620 cells (**H**) that were stably transfected with NY-ESO-1 expressing vectors (NY) or control vectors (CT) when they were grown in suspension conditions with or without PD98059 (50 μM) or trametinib (1 μM). **I**, **J** Representative FACS graphs (left) and statistical analysis (right) of cell death in A375 (**I**) and H1299 (**J**) cells that were stably transfected with NY-ESO-1-specific or control shRNAs when they were grown in suspension conditions with or without PD98059 (50 μM) or trametinib (2 μM).

Next, we investigated the mechanism through which NY-ESO-1 mediates anoikis resistance. By RNA-Seq analysis, we observed that NY-ESO-1 promoted activation of MAPK, especially ERK1/2 (Fig. 3A). ERK1/2 activity was previously reported to promote anoikis resistance and metastasis [27–29]. Thus, we investigated whether NY-ESO-1 exerts its functions by regulating ERK1/2 activation. We found that the levels of phospho-ERK1/2 increased when NY-ESO-1 was overexpressed. This effect was evident in HeLa, MCF-7, A375, MDA-MB-231, and SW620 cells (Fig. 3B). Conversely, when NY-ESO-1 was silenced in A375 and H1299 cells that naturally expressing NY-ESO-1, phospho-ERK1/2 decreased (Fig. 3C). Furthermore, when NY-ESO-1 was re-expressed in NY-ESO-1-knockdown cells, ERK1/2 activation was restored to levels seen in control cells (Fig. S2E). This function of NY-ESO-1 was also evident in cells cultured under suspension conditions. While ERK1/2 activation was decreased in suspended cells, NY-ESO-1 overexpression restored its activation (Fig. 3D). By contrast, knockdown of NY-ESO-1 noticeably reduced ERK1/2 activity in A375 cells cultured in suspension (Fig. 3E).

To assess the role of ERK1/2 activity in NY-ESO-1-mediated anoikis resistance, we used the MEK inhibitors trametinib and PD98059 to block ERK1/2 activation. Treatment with trametinib or PD98059 effectively eliminated the difference in apoptosis levels between cells with different levels of NY-ESO-1 expression (Fig. 3F–J, and Fig. S3). Furthermore, when trametinib was administered after control or NY-ESO-1-expressing MDA-MB-231 cells were inoculated in NCG mice via tail vein injection, it nearly completely prevented the lung metastasis of these cells (Fig. 2J). These results indicate that NY-ESO-1 promotes ERK1/2 activation, thereby suppressing anoikis and enhancing metastasis.

### NY-ESO-1 stabilizes PP1α by reducing its polyubiquitination

**Fig. 4:**
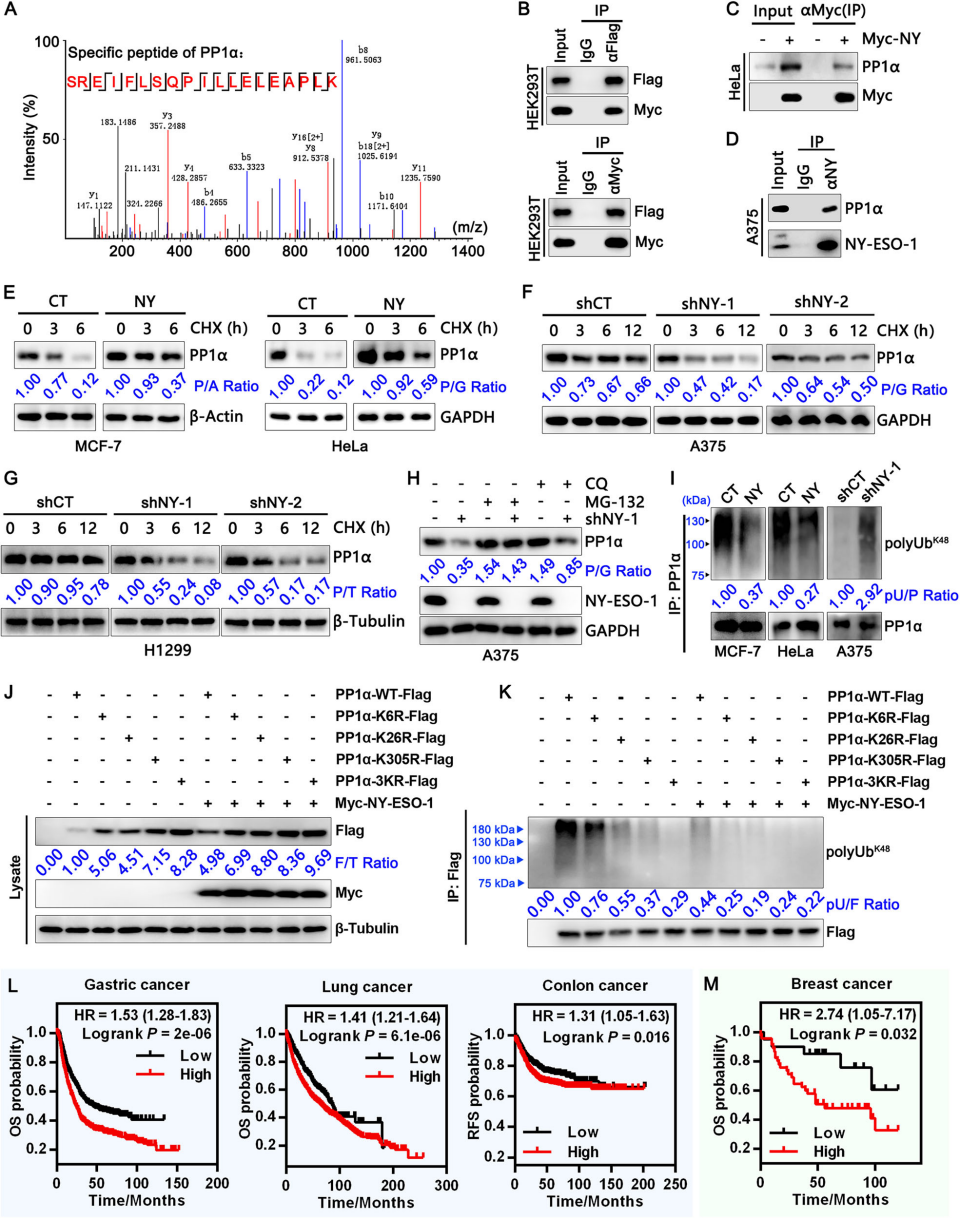
NY-ESO-1 interacts with and stabilizes PP1α by reducing its polyubiquitin modification. **A** Representative LC-MS/MS fragment spectrum of the PP1α-specific peptide. HEK293T cells were transfected with Myc-NY-ESO-1 plasmids and harvested 48 h later. Cell lysates were immunoprecipitated with anti-Myc antibodies, and the final immunoprecipitates were identified using LC-MS/MS. **B** Reciprocal co-immunoprecipitation of Myc and Flag in HEK293T cells co-transfected with Myc-NY-ESO-1 and Flag-PP1α vectors. **C** Immunoblot analysis of Myc-NY-ESO-1 immunoprecipitates from HeLa cells stably expressing NY-ESO-1 or control vectors indicated that NY-ESO-1 interacts with PP1α. **D** Endogenous interaction of NY-ESO-1 and PP1α in A375 cells. **E** Analysis of PP1α stability in MCF-7 (left) and HeLa cells (right) stably expressing NY-ESO-1 or control by the cycloheximide (CHX) chase assay. **F** CHX chase assay analysis of PP1α stability in A375 cells with stable NY-ESO-1 knockdown versus control. **G** CHX chase assay analysis of PP1α stability in H1299 cells with stable NY-ESO-1 knockdown versus control. (**H**) Immunoblot analysis of PP1α levels in stable NY-ESO-1-knockdown and control A375 cells with or without MG-132 (10 μM) or chloroquine (CQ, 50 μM) treatment. **I** Immunoblot analysis of PP1α ubiquitination in MCF-7 (left) and HeLa (middle) cells stably transfected with NY-ESO-1 or control vectors, or in A375 cells stably transfected with NY-ESO-1 shRNAs or control shRNAs (right). **J** Immunoblot analysis of the expression of PP1α mutants in HeLa cells co-transfected with NY-ESO-1 versus control vectors alongside indicated PP1α mutant vectors. **K** Immunoblot analysis of the ubiquitination of PP1α mutants in HeLa cells co-transfected with NY-ESO-1 (or control) vectors and the indicated PP1α mutant vectors. Cells were treated with MG132 for 4–6 h, then lysed with 1% SDS buffer and boiled for 5 min to denature. Subsequently, the lysates were diluted 10-fold with cold IP buffer and subjected to immunoprecipitation using anti-FLAG magnetic beads. For (**E**–**K**), P/A: PP1α/β-Actin, P/G: PP1α/GAPDH, P/T: PP1α/β-Tubulin, pU/P: polyUb^K48^/PP1α, F/T: Flag/β-Tubulin, pU/F: polyUb^K48^/Flag. **L** Survival curves were plotted for patients with gastric cancer (*n* = 875), lung cancer (*n* = 2166) and colon cancer (*n* = 1342) based on PP1α mRNA levels (200846_s_at). **M** The correlation between PP1α protein levels and survival probability in breast cancer (Tang_2018 dataset) was analyzed using the KM plotter. PP1α^low^: *n* = 20; PP1α^high^: *n* = 45.

To understand how NY-ESO-1 augments ERK1/2 activation, we examined a potential interaction between NY-ESO-1 and ERK1/2 or their regulators. Nevertheless, we observed that NY-ESO-1 did not interact with ERK1/2, their upstream kinases MEK1/2, or their key negative regulators DUSP2 and DUSP4 (Fig. S4). To identify novel NY-ESO-1-interacting proteins that may be involved in ERK signaling, we performed an anti-Myc-NY-ESO-1 immunoprecipitation (IP) assay coupled with LC-MS/MS. Of note, PP1α, a catalytic subunit of protein phosphatase 1 that was previously reported to regulate ERK1/2 activation [16], was identified as a potential interacting protein (Fig. 4A). Co-IP assays confirmed that Myc-NY-ESO-1 interacted with Flag-PP1α in HEK293T cells and that Myc-NY-ESO-1 interacted with endogenous PP1α in HeLa cells (Fig. 4B, C). Moreover, endogenous NY-ESO-1 and PP1α associated with each other in A375 cells (Fig. 4D).

To map the region of PP1α responsible for its interaction with NY-ESO-1, we generated several Flag-tagged PP1α deletion mutants (Fig. S5A) and transfected each of them together with full-length NY-ESO-1 into HEK293T cells. The region of PP1α comprising amino acid (aa) residues 7-230 was found to mediate the interaction with NY-ESO-1 (Fig. S5B). Likewise, we generated a panel of NY-ESO-1 mutations and found that the region encompassing aa 89-107 was important for the interaction with PP1α (Fig. S5C, D).

Forced NY-ESO-1 expression markedly increased PP1α stability and prolonged its half-life in MCF-7 and HeLa cells (Fig. 4E), while NY-ESO-1 knockdown promoted PP1α degradation and shortened its half-life in A375 and H1299 cells (Fig. 4F, G). Moreover, NY-ESO-1 knockdown noticeably decreased the protein level of PP1α, which could be rescued by the proteasome inhibitor MG-132 but not the autophagy inhibitor chloroquine (CQ) (Fig. 4H). Consistently, forced NY-ESO-1 expression reduced K48-linked poly-ubiquitination of PP1α in MCF-7 and HeLa, while NY-ESO-1 knockdown increased the levels of poly-ubiquitinated PP1α in A375 cells (Fig. 4I).

Proteome-wide analyses of endogenous ubiquitylation sites have identified three lysine residues on PP1α, K6, K26, and K305 (ref. [30, 31]). We mutated these residues individually or collectively to arginine. The K26R and especially K305R mutations showed a marked reduction in ubiquitination, while the K6R mutation showed a moderate reduction. The triple mutation, 3KR, nearly completely lost ubiquitination (Fig. 4J, K). PP1α 3KR was expressed at considerably higher levels than PP1α and was much less sensitive to the stabilizing effect of NY-ESO-1 (Fig. 4J, K). These results indicate that NY-ESO-1 stabilizes PP1α by preventing its ubiquitination and proteasomal degradation.

A survey of a public database showed that high PP1α mRNA levels correlated with poor prognoses in gastric, lung, and colon cancer patients (Fig. 4L). Moreover, breast cancer patients with high PP1α protein levels had much worse survival probability compared to those with low PP1α protein levels (Fig. 4M). These results suggest that PP1α promotes the progression of various cancers.

### PP1α is critical for NY-ESO-1-mediated ERK1/2 activation and anoikis resistance

**Fig. 5:**
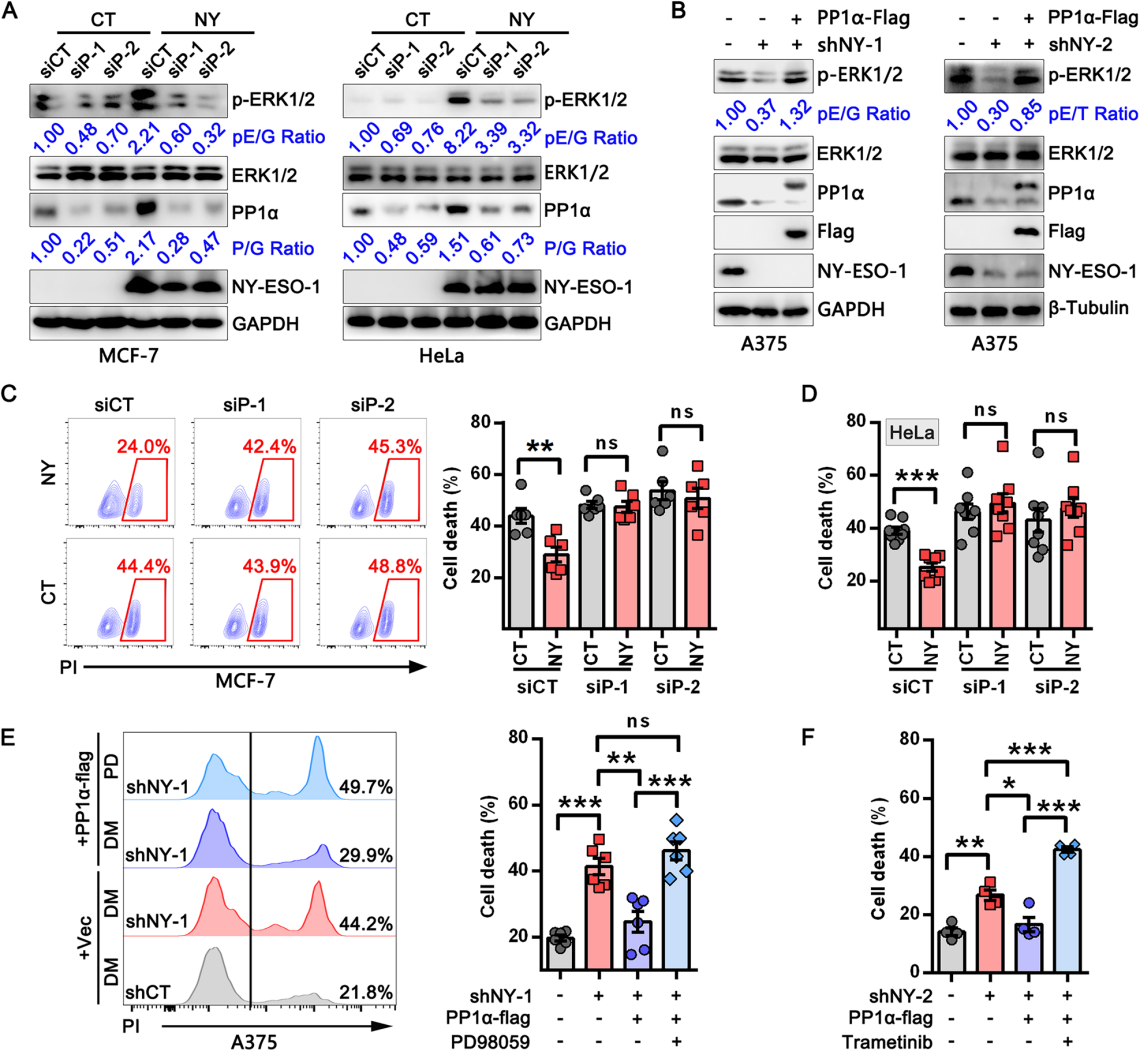
Silencing PP1α impairs NY-ESO-1-mediated ERK1/2 activation and anoikis resistance. **A** Immunoblot analysis of lysates from HeLa (left) and MCF-7 cells (right) stably expressing NY-ESO-1 or control, following transfection with control or PP1α siRNA for 48 h. **B** Immunoblot analysis of lysates from control, shNY-1 or shNY-2 stably expressing A375 cells with or without transient transfection of PP1α-flag. For (**A**, **B**), pE/G: pERK/GAPDH, pE/T: pERK/β-Tubulin, P/G: PP1α/GAPDH. **C**, **D** Representative FACS graphs (left) and statistical analysis (right) of cell death in NY-ESO-1 stably expressing MCF-7 (**C**), HeLa (**D**), and their corresponding control cells grown in suspension conditions with or without transient PP1α knockdown. **P < 0.01, ***P < 0.001, ns: not significant. **E**, **F** Representative FACS graphs (left) and statistical analysis (right) of cell death in shNY-1 or shNY-2 stably expressing A375 cells with or without indicated treatments, **P < 0.01, ***P < 0.001, ns: not significant.

PP1α is one of three catalytic subunits of protein phosphatase 1 (PP1) and has been reported to promote ERK1/2 activation by modulating B-Raf activity or enhancing Raf-1/14-3-3 interactions [16, 32]. Consistent with this role, PP1α knockdown in wild-type cancer cells significantly reduced the activity of ERK1/2 and its upstream regulators MEK1/2, B-Raf, and C-Raf (Raf-1) (Fig. S6A), confirming that PP1α activates ERK1/2 signaling through Raf-dependent mechanisms. We therefore speculated that PP1α is required for NY-ESO-1-mediated ERK1/2 activation. To test this, we knocked down PP1α in NY-ESO-1-overexpressing cells. While PP1α knockdown had only a minor effect in control cells, it significantly reduced ERK1/2 phosphorylation in NY-ESO-1-overexpressing cells (Fig. 5A). We also overexpressed PP1α in NY-ESO-1-knockdown A375 cells and observed an effective restoration of ERK1/2 activity (Fig. 5B), indicating that PP1α acts downstream of NY-ESO-1 to promote ERK1/2 activation.

Consistently, NY-ESO-1 overexpression did not alter the extent of anoikis in PP1α-knockdown cells (Fig. 5C, D). By contrast, PP1α overexpression in NY-ESO-1 knockdown cells could significantly reduce anoikis, and this protective effect vanished when the ERK1/2 pathway was blocked by PD98059 or trametinib (Fig. 5E, F). Moreover, full-length or truncated mutants of NY-ESO-1 with the ability to interact with PP1α were able to promote ERK1/2 activation and protect cancer cells from death in suspension, whereas aa1-88 truncate, which cannot interact with PP1α, was unable to do so (Fig. S6B, C, Fig. S1I and Fig. S2E). These findings suggest that PP1α is indispensable for ERK1/2 activation and anoikis resistance mediated by NY-ESO-1.

### NY-ESO-1 engages OTUB1 to promote deubiquitination of PP1α

Although NY-ESO-1 inhibits polyubiquitination of PP1α, it does not contain a deubiquitinase (DUB) domain or share any discernable sequence similarity with DUB domains (https://pfam.xfam.org/) [33]. A recent study suggested that USP11 can stabilize PP1α by deubiquitinating it and protecting it from proteasomal degradation in colorectal cancer [34]. However, although NY-ESO-1 was associated with USP11, it did not significantly alter the USP11-PP1α interaction (Fig. S7A, B). Critically, USP11 knockdown abrogated neither NY-ESO-1-mediated PP1α stabilization (Fig. S7C) nor NY-ESO-1-conferred protection against anoikis (Fig. S7D–G).

**Fig. 6:**
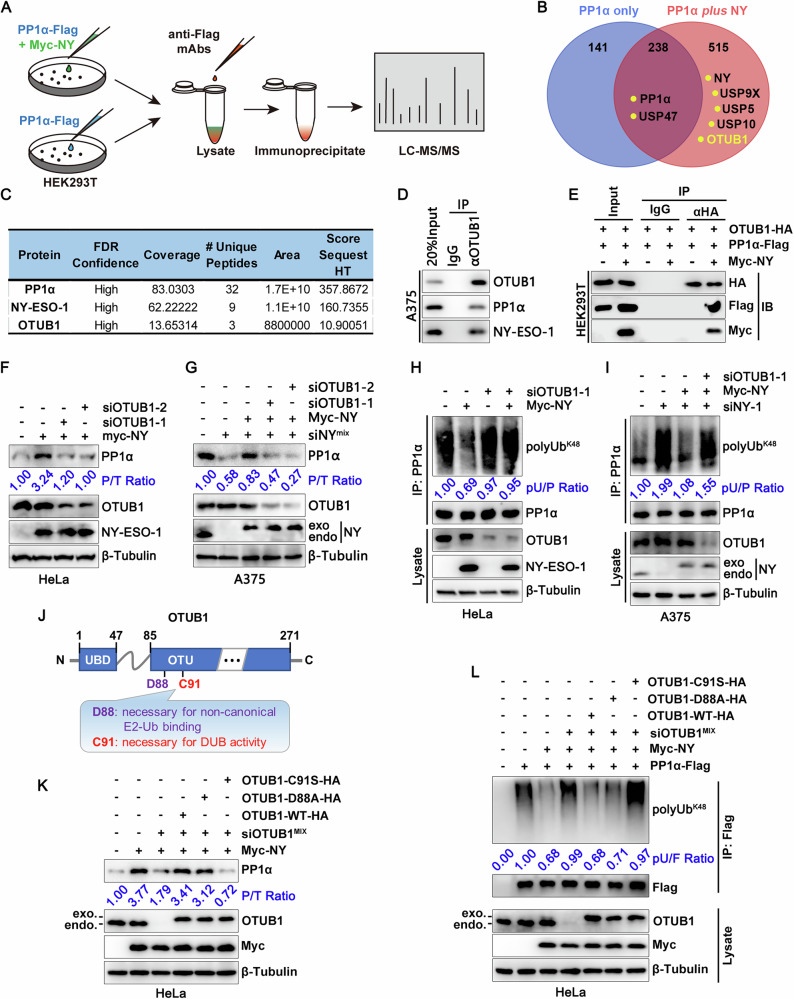
OTUB1 participates in the interaction between NY-ESO-1 and PP1α and is required for NY-ESO-1-mediated PP1α deubiquitination. **A** Schematic procedure of PP1α interactome analysis by co-IP coupled with LC-MS/MS assay. HEK293T cells were transfected with PP1α-Flag alone or in combination with Myc-NY-ESO-1 and harvested 48 h later. Cell lysates were immunoprecipitated with anti-Flag antibodies, and the final immunoprecipitates were identified using LC-MS/MS. **B** Venn diagram showing the effect of NY on PP1α interactome. **C** A table listing the interactive proteins identified by the LC-MS/MS assay along with the corresponding parameters. **D** Endogenous interaction of NY-ESO-1, OTUB1 and PP1α in A375 cells. **E** Immunoblot analysis of the interaction between NY-ESO-1, PP1α, and OTUB1 in HEK293T cell lysates following IP with anti-HA mAbs. **F** Immunoblot analysis of the indicated proteins in HeLa cells transfected with NY-ESO-1 plasmids together with two additional individual siRNAs targeting OTUB1 or control siRNAs. **G** Immunoblot analysis of the indicated proteins in A375 cells transfected with a mixture of NY-ESO-1 siRNAs (siNY-1:siNY-2, 1:1) along with OTUB1 siRNAs (siOTUB1-1 or siOTUB1-2) and/or NY-ESO-1 plasmids. **H**, **I** Immunoblot analysis of PP1α ubiquitination in HeLa cells (**H**) and A375 cells (**I**) post indicated transfections. **J** Schematic of the domain architecture of OTUB1, showing its OTU domain, and ubiquitin-binding domain (UBD), with specific mutations in residues that were used in this study. **K** Immunoblot analysis of the indicated proteins in HeLa cells transfected with NY-ESO-1 plasmids together with a mixture of OTUB1 siRNAs (siOTUB1-1:siOTUB1-2, 1:1) and wild-type or mutated OTUB1 plasmids. **L** Immunoblot analysis of PP1α ubiquitination in HeLa cells post indicated transfections. Cells were treated with MG132 for 4–6 h, then lysed with 1%SDS buffer and boiled for 5 min to denature. Subsequently, the lysates were diluted 10-fold with cold IP buffer and subjected to immunoprecipitation using anti-FLAG magnetic beads. P/T PP1α/β-Tubulin, pU/P polyUb^K48^/PP1α, F/T Flag/β-Tubulin, pU/F polyUb^K48^/Flag.

To understand how NY-ESO-1 promotes PP1α deubiquitination, we immunoprecipitated PP1α protein from lysates of HEK293T cells expressing PP1α-Flag alone or in combination with Myc-NY-ESO-1 and analyzed immunoprecipitates by LC-MS/MS to determine the difference in PP1α interactomes (Fig. 6A). We discovered 515 putative binding proteins that are unique to cells that express NY-ESO-1 (Fig. 6B), among which were several DUBs, including USP47, USP9X, USP5, USP10, and OTUB1 (Fig. 6B, C). Knocking down each of the four USPs had a minimal effect on the regulation of PP1α by NY-ESO-1 (Fig. S8). Of note, OTUB1 interacted with both NY-ESO-1 and PP1α at levels of endogenous proteins (Fig. 6D). Its interaction with PP1α was mediated by NY-ESO-1, as it showed no binding to PP1α in the absence of NY-ESO-1 (Fig. 6E). Domain mapping assays showed that the region encompassing aa 47–159 of OTUB1 and the region encompassing aa 89-107 of NY-ESO-1 were required for their interaction (Fig. S9). OTUB1 knockdown using two independent siRNAs rendered NY-ESO-1 incapable of increasing PP1α abundance (Fig. 6F, G) and eliminated NY-ESO-1-mediated anoikis resistance (Fig. S10). This functional distinction from USP11 knockdown identifies OTUB1 as essential for NY-ESO-1-dependent stabilization of PP1α and promotion of anoikis resistance. Mechanistically, OTUB1 modulates PP1α ubiquitination, and its ablation effectively abrogated the inhibitory effect of NY-ESO-1 on PP1α polyubiquitination (Fig. 6H). Moreover, while forced expression of NY-ESO-1 reduced PP1α ubiquitination in A375 cells treated with NY-ESO-1 siRNA, it failed to do so in those treated with both NY-ESO-1 and OTUB1 siRNAs (Fig. 6I). OTUB1 is an enzyme that cleaves polyubiquitin chains connected to Lys-48; it also performs a non-catalytic role in ubiquitylation suppression by binding E2 enzymes [35]. To investigate how OTUB1 regulates PP1α stability, we created a catalytic mutant (C91S) and an E2-interacting mutant (D88A) of OTUB1 (Fig. 6J). Surprisingly, the C91S mutant lost its capacity to interact with NY-ESO-1, but the D88A mutant continued to do so (Fig. S11). Furthermore, in OTUB1-knockdown cells, re-expression of the wild-type OTUB1 and D88A mutant, but not the catalytically inactive C91S mutant, significantly increased PP1α expression and decreased the amounts of K48-polyubiquitin chains of PP1α (Fig. 6K, L). These findings imply that OTUB1’s deubiquitinase activity, rather than its capacity to bind E2, is necessary for suppressing Lys48-linked PP1α polyubiquitination and improving PP1α stability. Taken together, these results indicate that NY-ESO-1 facilitates OTUB1-mediated PP1α deubiquitination by acting as a scaffold protein to facilitate their interaction.

## Discussion

**Fig. 7:**
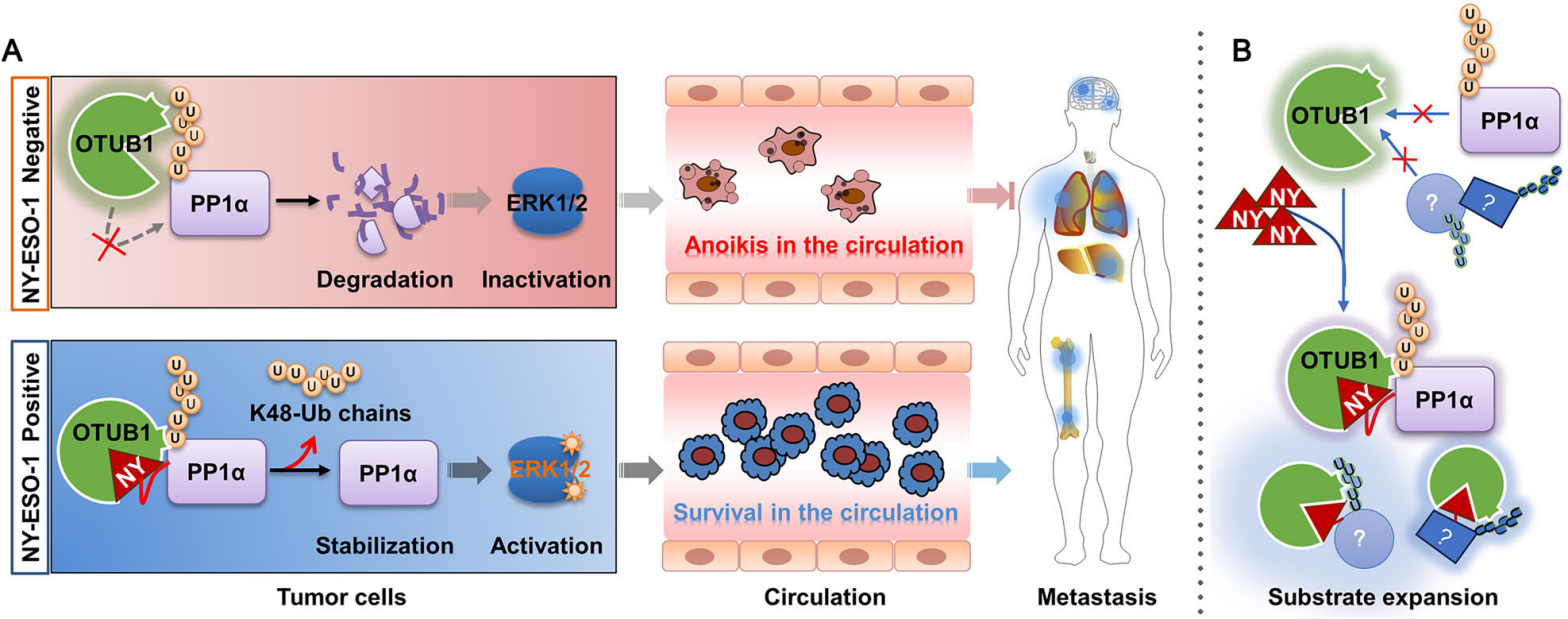
Schematic model depicting NY-ESO-1-mediated anoikis resistance and tumor metastasis, as well as substrate expansion for OTUB1. **A** NY-ESO-1 interacts with PP1α and employs the deubiquitinase OTUB1 to reduce its polyubiquitin modification. This leads to PP1α protein stabilization, enhanced ERK1/2 activation, and consequently, increased anoikis resistance and tumor metastasis. **B** NY-ESO-1 acts as an adaptor protein that recruits unconventional substrates, such as PP1α, to OTUB1 or other deubiquitinases like USP11, USP9X, and USP5. This results in the formation of the DUB complex, which effectively prevents these unconventional substrates from being ubiquitinated.

NY-ESO-1 is a promising therapeutic target, but its unclear biological function has hindered the development of effective targeted therapies. In this study, we demonstrate that NY-ESO-1 is associated with poor prognosis in cancer patients, particularly those with immune-cold tumors. We further investigate its role in anoikis resistance and tumor metastasis, and uncover the underlying mechanisms. Specifically, we identify a novel signaling axis in which NY-ESO-1 recruits the deubiquitinase OTUB1 to prevent PP1α ubiquitination and degradation, thereby enhancing ERK1/2 activation (Fig. 7A). This finding has important implications in the development of effective strategies for preventing tumor metastasis, which may be more effective against NY-ESO-1-driven tumors when used alone or in combination with other strategies. While current NY-ESO-1-targeted strategies primarily focus on immunotherapy, either by directly targeting it through TCR-based therapies or by leveraging its immunogenic properties with cancer vaccines [36], our findings introduce an alternative therapeutic approach that targets the function of NY-ESO-1 in tumor progression. This strategy has the potential to expand treatment options for patients with NY-ESO-1-positive tumors.

Metastasis represents the most deadly aspect of cancer and is responsible for 90% of cancer deaths [2, 37]. Current treatments for cancer metastasis are mainly chemotherapy and radiotherapy [2], both of which are insufficient and ineffective due to their side effects and drug resistance [38–40]. Anoikis resistance allowing tumor cells to survive during spread and circulation, is a prerequisite for successful metastasis, and has been recognized as an attractive therapeutic target [4, 28]. Many signaling pathways (e.g., ERK1/2, NF-κB, and Akt) are involved in anoikis resistance and can be efficiently targeted [8, 28]; however, they also play an essential role in normal cell survival and functions. Thus, it is hard to completely block such pathways without side effects. Our data uncover a previously unrecognized role of NY-ESO-1 in anoikis resistance. As NY-ESO-1 knockdown promotes anoikis and impairs tumor metastasis, NY-ESO-1 may be essential for metastatic cancer cells. Given its restricted expression pattern, NY-ESO-1 may be a more attractive and promising target for preventing metastasis.

The biological function of NY-ESO-1 remains incompletely understood. Previous studies have shown that treatment with epigenetic modulators, such as histone deacetylase inhibitors and DNA demethylating agents, induces NY-ESO-1 expression and leads to cancer cell death [41]. However, this cytotoxic effect appears to be independent of NY-ESO-1 itself, as ectopic expression does not affect cell viability [41]. In contrast, U266 myeloma cells, which grow in suspension and express high levels of endogenous NY-ESO-1, exhibit significantly reduced colony formation, adriamycin resistance, and invasive capacity in vitro upon NY-ESO-1 knockdown, alongside decreased tumor growth and reduced osteolytic lesions in vivo [42]. Consistent with these reports, we demonstrate that NY-ESO-1 modulation in adherent cells neither alters apoptotic levels nor impacts proliferation under normal conditions; however, under suspension-induced stress, NY-ESO-1 knockdown significantly increases sensitivity to anoikis and reduces colony formation, while its overexpression suppresses anoikis and promotes colony formation. These results suggest that NY-ESO-1 is dispensable for cell survival and proliferation under homeostatic conditions but critically required for malignant stress adaptation (e.g., detachment), redefining its role in tumor pathobiology.

PP1α, an isoform of serine/threonine-protein specific phosphatase encoded by the *PPP1CA* gene [43], is implicated in a large variety of cellular functions by dephosphorylating its substrates [16, 34, 44, 45]. PP1α is overexpressed in a variety of tumors and cancer cell lines, yet its role in tumor progression remains ambiguous [46, 47]. PP1α was previously shown to suppress oncogenic growth by activating Rb, caspase 9, and BAD through dephosphorylation [43, 48–52]. In contrast, recent studies reported that PP1α overexpression activates the MAPK pathway and promotes cancer cell invasiveness [16, 34]. Our results further confirmed such metastasis-promoting function of PP1α and identified NY-ESO-1 as its novel regulator. NY-ESO-1 binds to PP1α and reduces its ubiquitination and degradation by recruiting the deubiquitinating enzyme OTUB1, leading to PP1α accumulation and subsequent ERK1/2 activation. Importantly, knockdown or loss-of-function mutation of OTUB1 abrogated NY-ESO-1–mediated stabilization of PP1α and diminished its ability to facilitate anoikis resistance. Although USP11 has previously been reported to stabilize PP1α [34] and was found to interact with both NY-ESO-1 and PP1α in our system, its knockdown did not affect PP1α levels or NY-ESO-1-dependent anoikis resistance. The distinct effects of OTUB1 and USP11 knockdown highlight OTUB1 as the critical functional mediator of NY-ESO-1 activity. While the precise molecular details of the interactions among NY-ESO-1, OTUB1, and PP1α remain to be fully elucidated, our data provide the first evidence that NY-ESO-1 functions as a novel regulator of PP1α and plays a key role in PP1α-driven tumor metastasis.

Moreover, our findings reveal a previously unanticipated pattern of deubiquitinase substrate expansion. As an adaptor protein, NY-ESO-1 recruits OTUB1 and PP1α, which are not meant to interact directly, to form the DUB complex, which prevents PP1α from being ubiquitinated (Fig. 7B). In addition to OTUB1, NY-ESO-1 can interact with additional DUBs such as USP11, USP9X, USP5, and others. While we do not yet know what pairing substrates they respond to, it is possible that broadening the range of substrates via adaptors is a general mechanism (Fig. 7B). This discovery opens new avenues for investigating the underlying mechanism of DUB substrate selection and advancing the development of medicines targeting DUB signaling pathways.

In summary, our study demonstrates a new function of NY-ESO-1 that mediates anoikis resistance and tumor metastasis through OTUB1-mediated PP1α stabilization and subsequent ERK1/2 activation. These findings suggest that NY-ESO-1 is not only a target for tumor immunotherapy, but also plays a key role in tumor progression and may be a target for preventing tumor metastasis, which may offer new paths and emphasis for the development of NY-ESO-1-based therapeutic strategies.

## Data and materials availability

All relevant datasets supporting the conclusions of this study are available within the article and its additional files.

## Supplementary information

Supplementary Materials

Table S2. Lists of proteins identified by LC-MS_MS

Table S3. Lists of genesets enriched in NY-ESO-1

Original data
