# Supplementary material for: NY-ESO-1 facilitates anoikis resistance and tumor metastasis by hijacking deubiquitinase OTUB1 to stabilize PP1α
Source: Cell Death Dis. 2025 Oct 6;16(1):682. doi: 10.1038/s41419-025-08017-w (PMC12500907; doi:10.1038/s41419-025-08017-w)
Supplement: Supplementary file 1 — Supplementary Materials [file 41419_2025_8017_MOESM1_ESM.docx]

Supplementary Materials for

**NY-ESO-1 facilitates anoikis resistance and tumor metastasis by hijacking deubiquitinase OTUB1 to stabilize PP1α**

Pengchao Zhang, *et al.*

*Corresponding author. Guizhong Zhang, gz.zhang@siat.ac.cn

Xiaolu Yang, [xyang@pennmedicine.upenn.edu](mailto:xyang@pennmedicine.upenn.edu)

Xiaochun Wan, xc.wan@siat.ac.cn

**This PDF file includes:**

Supplementary Text

Figs. S1 to S11

Tables S1 to S4

References (1 to 8)

Supplementary Methods

**Cell viability and proliferation assay**

The impact of NY-ESO-1 on cell viability was determined using an MTS assay (CellTiter 96® Aqueous One Solution Cell Proliferation Assay, Promega) according to the manufacturer's instructions.

Cell proliferation was detected by EdU incorporation assay (EdU Cell Proliferation Kit with Alexa Fluor 647, Beyotime). In short, cells were plated in triplicates at 1.0×10^5^ cells/well in either normal or poly-HEMA-coated 12-well plates, and then kept in a medium containing 10% FBS for an additional 6 hours in poly-HEMA-coated plates, or overnight in normal plates. Following two hours of incubation at 37°C with 10 μM EdU, the cells were harvested, fixed with 4% Paraformaldehyde, and permeabilized with 0.3% Triton X-100. The newly synthesized DNA incorporated with EdU was then fluorescently labeled by adding the reaction mix to the cells and incubating them for 30 minutes at room temperature. The EDU-positive proliferating cell fractions were identified using FACS.

**Transwell migration assay**

Cell migration assays were performed using BD Falcon Cell Culture Inserts (BD, San Jose, CA). Cells were pre-incubated in the serum-free medium overnight. 1 × 10^5^ cells were placed in the insert and allowed to migrate for 18 h. The outer chamber was filled with 600 μL of medium containing 10% FBS. After incubation, non-migrating cells on the upper surface of the insert were removed with a cotton swab. Migrated cells were fixed and stained with crystal violet, imaged, and then eluted with 10% acetic acid. Absorbance was measured at 595 nm using a microplate reader.

**RNA isolation and qRT-PCR**

Total RNA was extracted using TRIzol reagent (Invitrogen) and used to generate cDNA. Specific primers used for quantitative real-time PCR assays were synthesized by GENEWIZ, Inc. (Suzhou, China). The primer sequences are provided in Table S4, while the raw data are provided in the supplemental file titled “original data”.


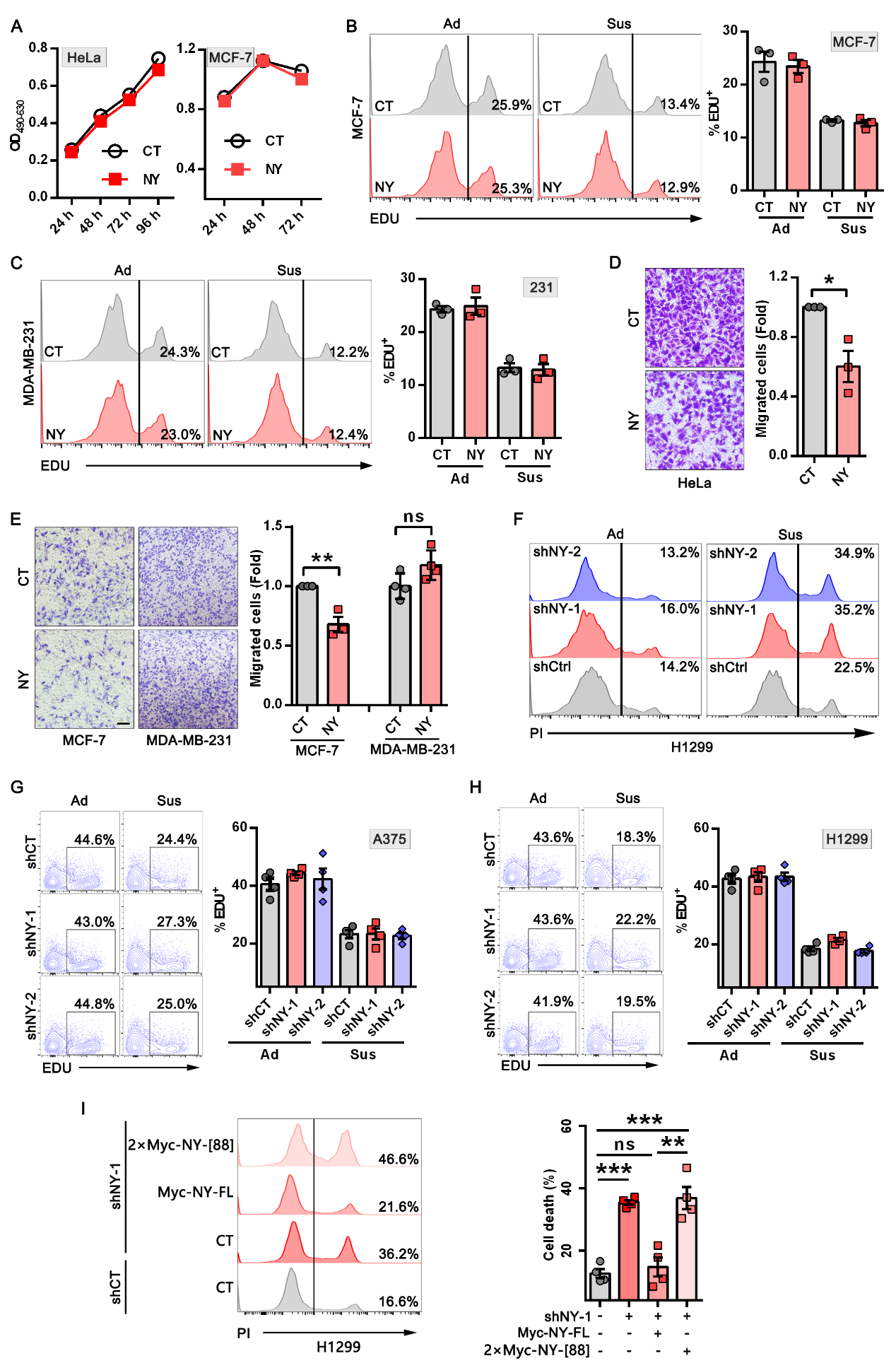


**Figure S1. Effect of NY-ESO-1 on tumor cell proliferation, migration, apoptosis and signaling transduction**

(A) The cell proliferation of control and NY-ESO-1 stably expressing HeLa cells and MCF-7 cells. (B and C) FACS-based detection of EDU-positive fractions of indicated MCF-7 and MDA-MB-231 cells in either adherent or suspension. (D and E) Representative images and quantitation of migrated HeLa (D), MCF7, and MDA-MB-231 (E) cells in the Transwell migration assay. CT: control vectors, NY: NY-ESO-1 expressing vectors. **P*＜0.05, ***P*＜0.01, ns: not significant. (F) Representative FACS graphs of cell death in the indicated H1299 cells cultured in either adherent or suspension. (G and H) Representative photographs (left) and statistical analysis (right) of EDU-positive fractions of indicated A375 (G) and H1299 cells (H) in either adherent or suspension. (I) FACS-based analysis of anoikis in H1299 cells with or without indicated transfections. ***P*＜0.01, ****P*＜0.001.


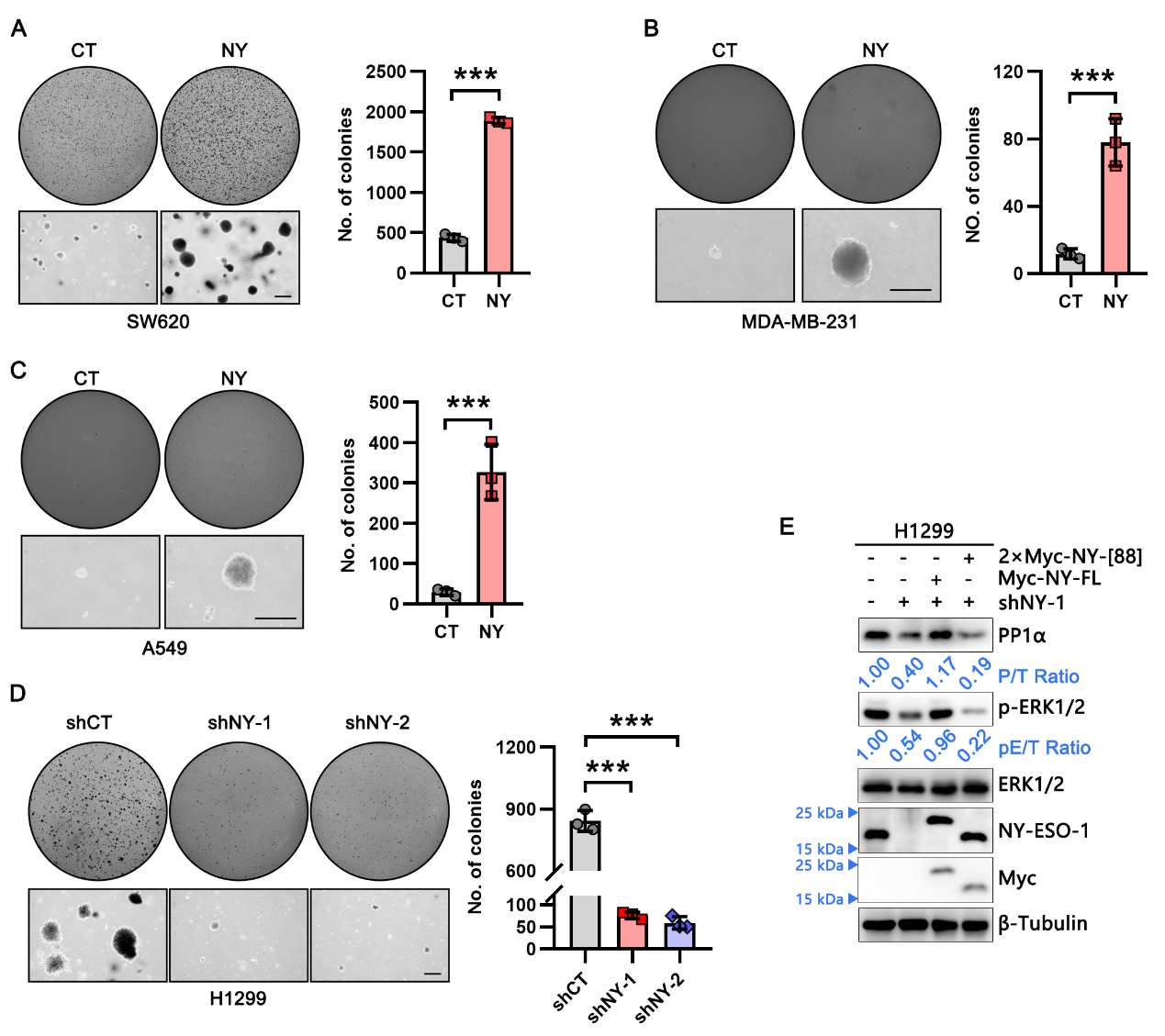


**Figure. S2 Effect of NY-ESO-1 on colony formation in soft agar and signaling transduction**

(A-C) Soft agar colony formation assays comparing NY-ESO-1-expressing SW620 (A), MDA-MB-231 (B), and A549 (C) cells to their respective controls. Left panels: Representative photographs. Right panels: Statistical analysis of colony numbers. Scale bar = 500 μm. ****P*＜0.001. (D) Soft agar colony formation by control and NY-ESO-1-knockdown A375 cells. Left: Representative images. Right: Colony quantification. Scale bar = 500 μm, ****P*＜0.001. (J) Immunoblot analysis of ERK1/2 phosphorylation and PP1α expression in the control or shNY-1 stably expressing H1299 cells with or without transfection of Myc-NY-ESO-1-FL or 2×Myc-NY-[1-88]. P/T: PP1α/β-Tubulin, pE/T: pERK/β-Tubulin.


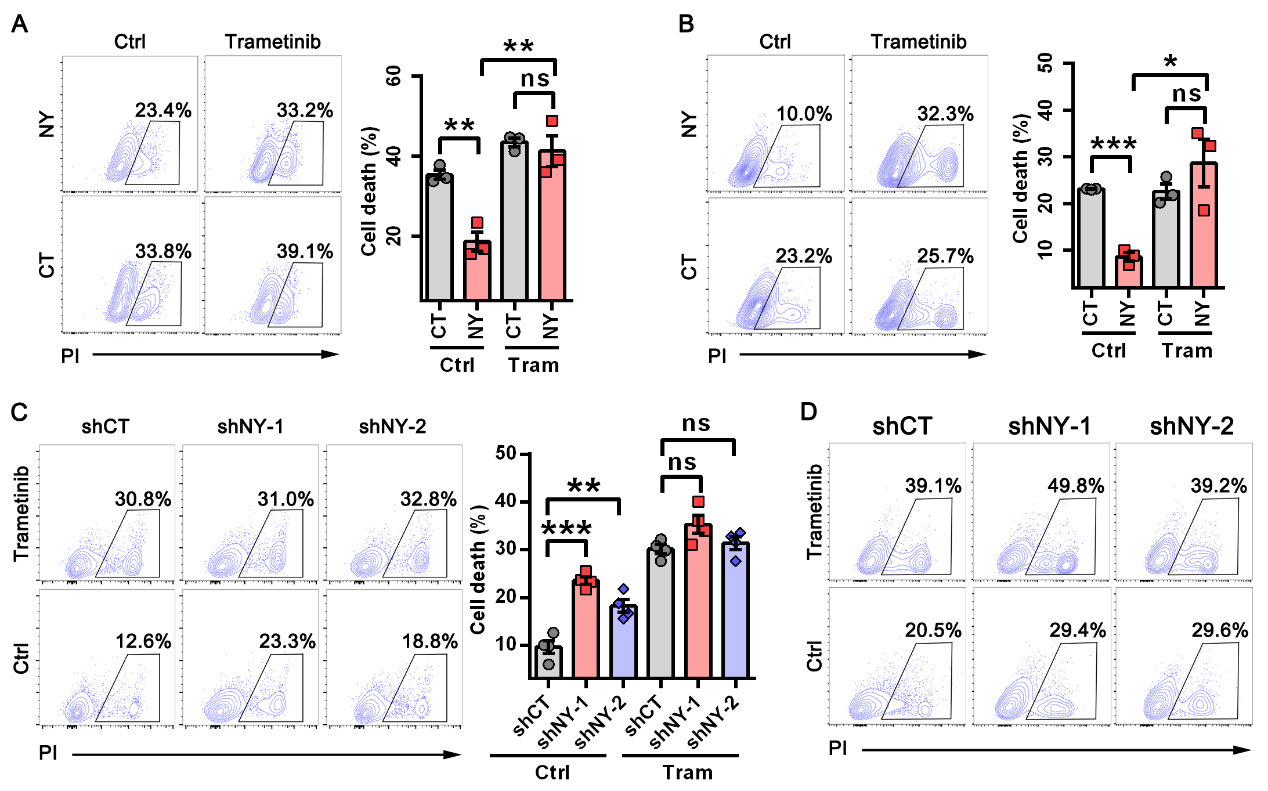


**Figure S3. Blockade of MEK-ERK1/2 pathway eliminates NY-ESO-1 function in regulating cell death**

(A and B) FACS-based analysis of cell death in NY-ESO-1 overexpressing and control MCF-7 (A) and MDA-MB-231 (B) cells grown in suspension conditions with or without the MEK inhibitor trametinib (1μM). Representative FACS graphs (left) and quantification (right) are shown. (C) FACS-based analysis of cell death in NY-ESO-1 knockdown and control A375 cells grown in suspension conditions with or without trametinib treatment (2 μM). (D) Representative FACS graphs of cell death in NY-ESO-1 knockdown and control H1299 cells grown in suspension conditions with or without trametinib treatment (2 μM).


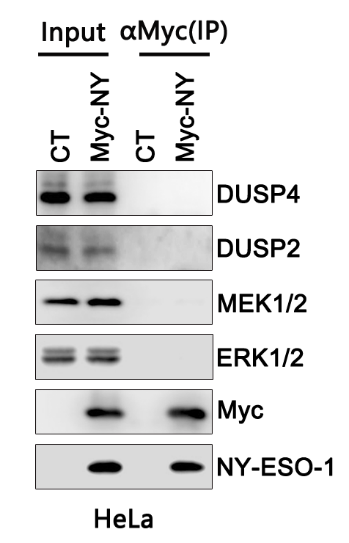


**Figure S4. Interactions of NY-ESO-1 with proteins involved in ERK1/2 pathway.**

Immunoblot analysis of Myc-NY-ESO-1 immunoprecipitates from HeLa cells stably expressing NY-ESO-1 or control vectors.


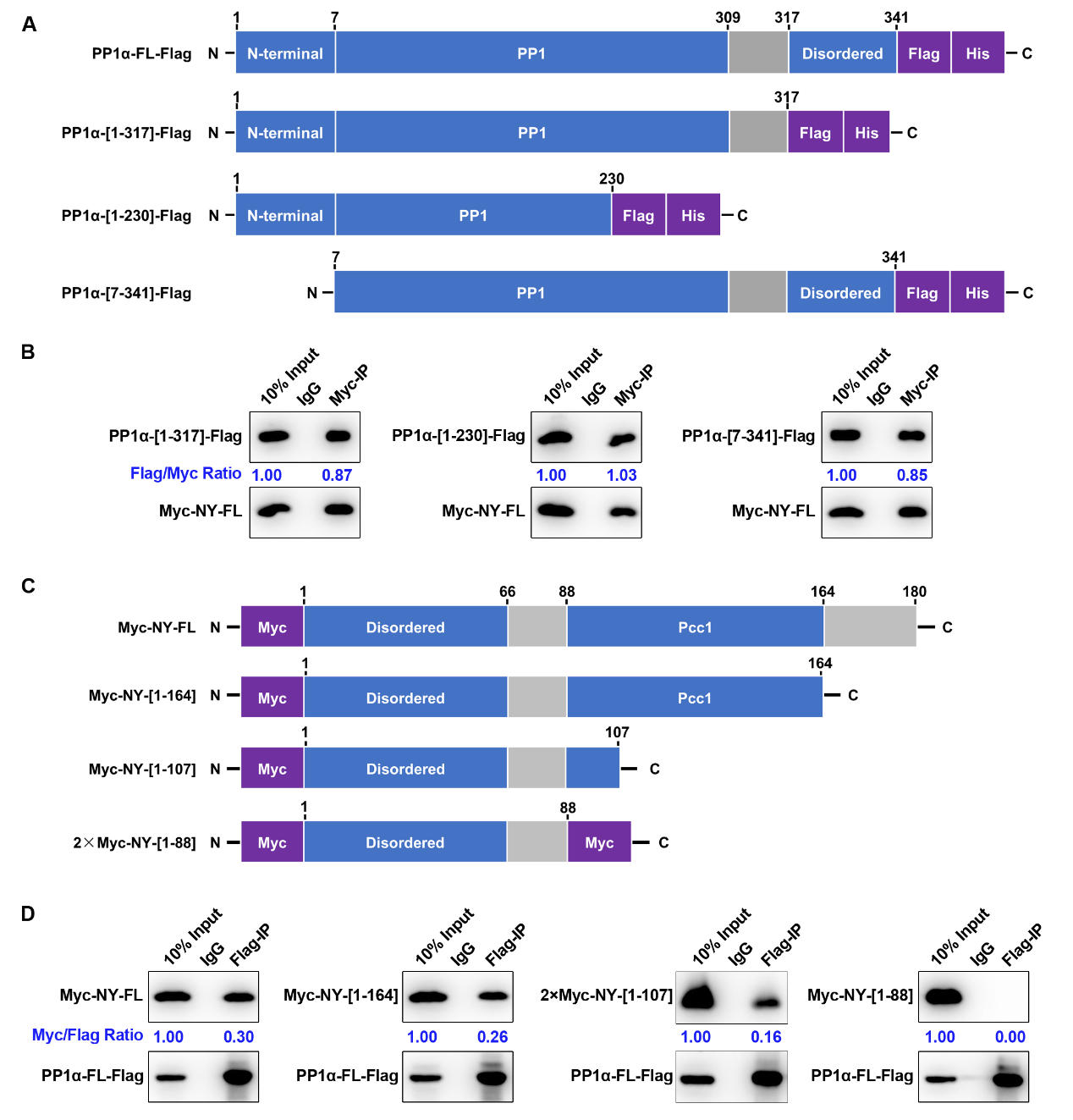


**Figure S5. Domain mapping of the interaction between NY-ESO-1 and PP1α**

(A) Schematic diagram of Flag-tagged PP1α deletion mutants used for the interaction assay. (B) Immunoprecipitations (IP) using anti-Myc antibodies from HEK293T cells transfected with Myc-NY-ESO-1 plasmids along with Flag-tagged PP1α deletion mutants as indicated. (C) Schematic representation of Flag-tagged PP1α deletion mutants. (D) Immunoblot analysis of the interactions between full-length PP1α with indicated NY-ESO-1 truncates in HEK293T cell lysates after IP with anti-Flag mAbs.


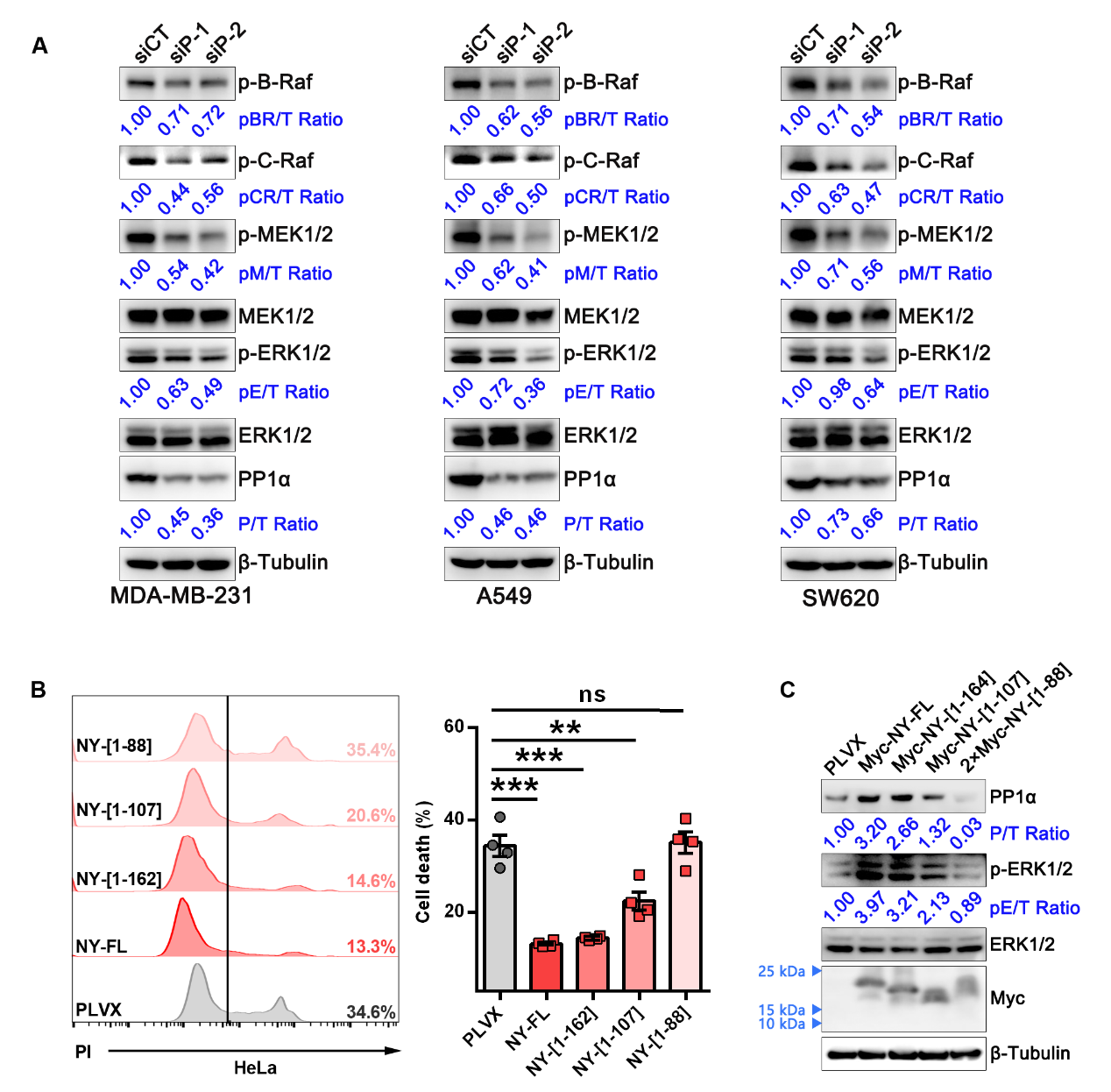


**Figure S6. Role of PP1α in regulating the ERK1/2 signaling pathway and function verification of NY-ESO-1/PP1α interaction**

(A) Western blot analysis of ERK1/2 pathway activation in PP1α-knockdown cells. Phosphorylation status of ERK1/2 and upstream regulators MEK1/2, C-Raf, and B-Raf was compared between control and PP1α siRNA-transfected MDA-MB-231, A549, and SW620 cells (left to right). (B) FACS-based analysis of anoikis in HeLa cells with or without indicated transfections. (C) The ERK1/2 phosphorylation and PP1α expression of HeLa cells transiently transfected with indicated plasmids. P/T: PP1α/β-Tubulin, pE/T: pERK/β-Tubulin. pBR/T: p-B-Raf/β-Tubulin, pCR/T: p-C-Raf/β-Tubulin, pM/T: p-MEK/β-Tubulin, pE/T: p-ERK/β-Tubulin, P/T: PP1α/β-Tubulin.


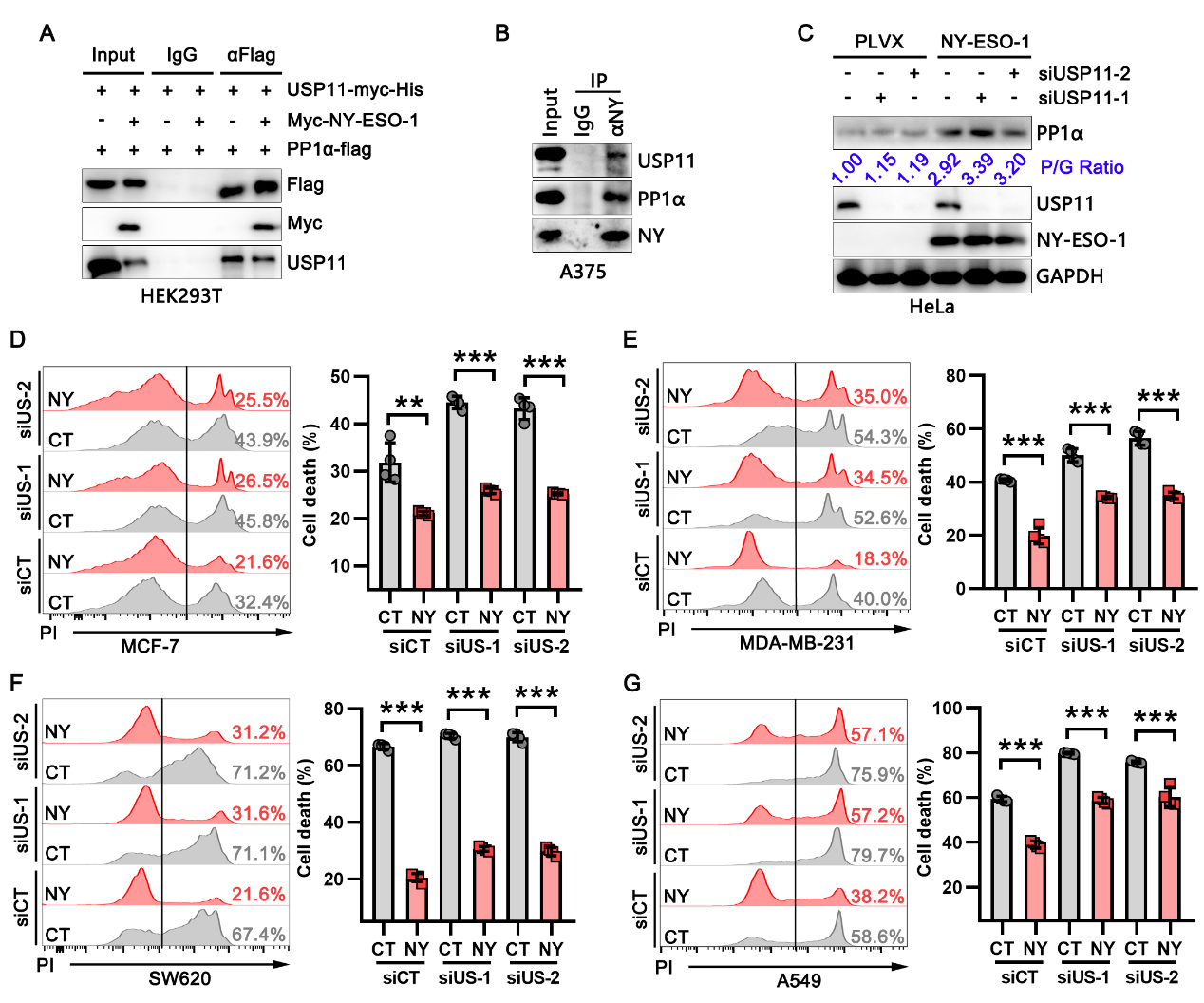


**Figure S7. Role of USP11 in NY-ESO-1-mediated PP1α regulation and anoikis resistance**

(A) Immunoblot analysis of the interaction between NY-ESO-1, PP1α, and USP11 in HEK293T cell lysates after IP with anti-Flag mAbs. (B) Immunoblot analysis of the endogenous interaction between NY-ESO-1, PP1α, and USP11 in A375 cells. Cell lysates were immunoprecipitated by anti-NY-ESO-1 mAbs and analyzed by immunoblot. (C) Immunoblot analysis of PP1α protein levels in A375 cells transfected with USP11 siRNAs. P/G: PP1α/GAPDH. (D-G) Cell death analysis under suspension culture after OTUB1 knockdown in NY-ESO-1-expressing and control cells. Representative FACS graphs (left) and statistical analysis (right) of cell death in MCF-7 (D), MDA-MB-231 (E), SW620 (F), and A549 cells (G) are shown. Cells stably expressing control vector (CT) or NY-ESO-1 (NY) were transfected with USP11-targeting siRNAs (siUS-1, siUS-2), cultured in suspension for 72-120 h, and analyzed by PI staining. ***P*＜0.01; ****P*＜0.001.


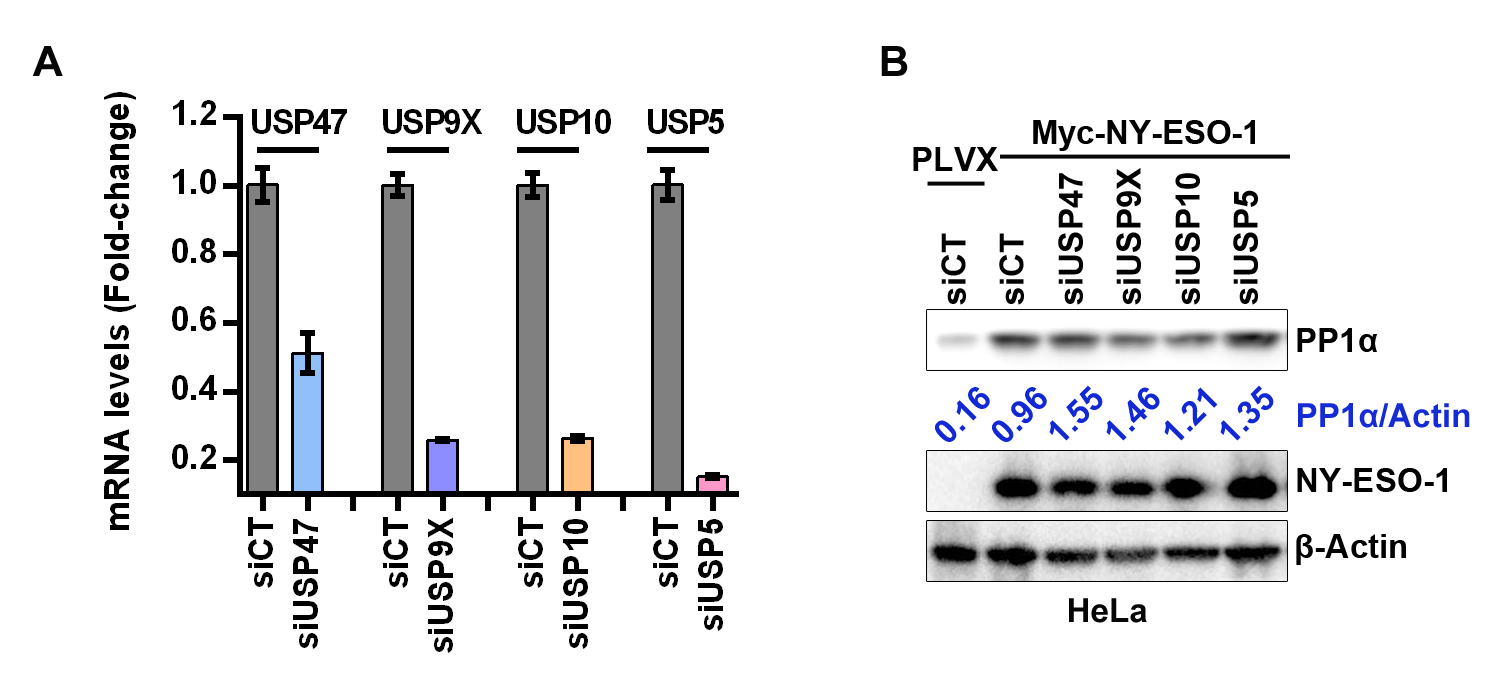


**Figure S8. Lack of a role for USPs in NY-ESO-1-mediated PP1α regulation**

(A) Real-time PCR analysis of USP47, USP9X, USP10, and USP5 mRNA levels in HeLa cells transfected with corresponding siRNAs. (B) Immunoblot analysis of PP1α protein levels in HeLa cells transfected with the indicated USPs siRNAs.


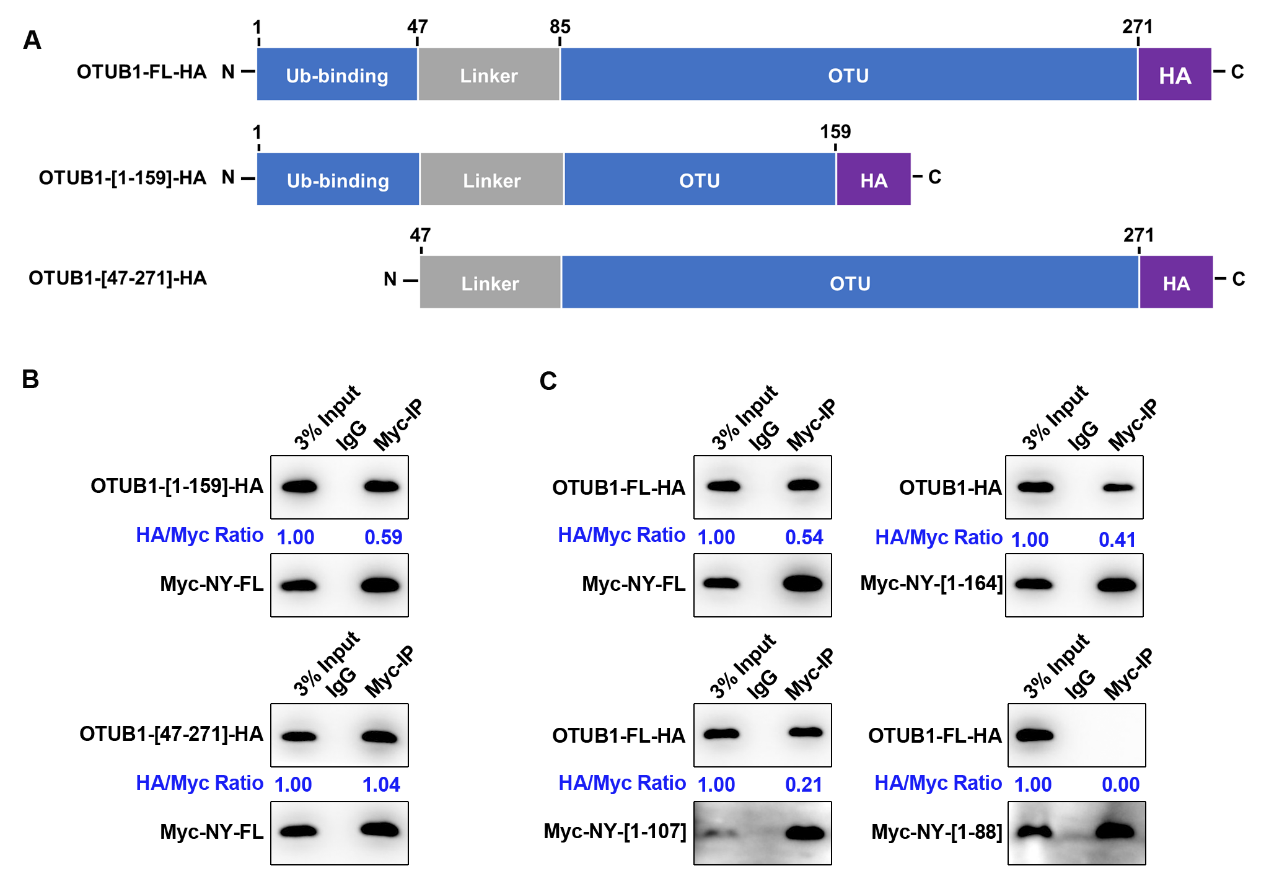


**Figure S9. Domain mapping of the interaction between NY-ESO-1 and OTUB1**

(A) Schematic diagram of HA-tagged OTUB1 truncated mutants used for the interaction assay. (B) Immunoprecipitations showing the interactions between full-length NY-ESO-1 and indicated OTUB1 mutants in HEK293T cells. (C) Immunoprecipitations showing the interactions between full-length OTUB1 and indicated NY-ESO-1 mutants in HEK293T cells.


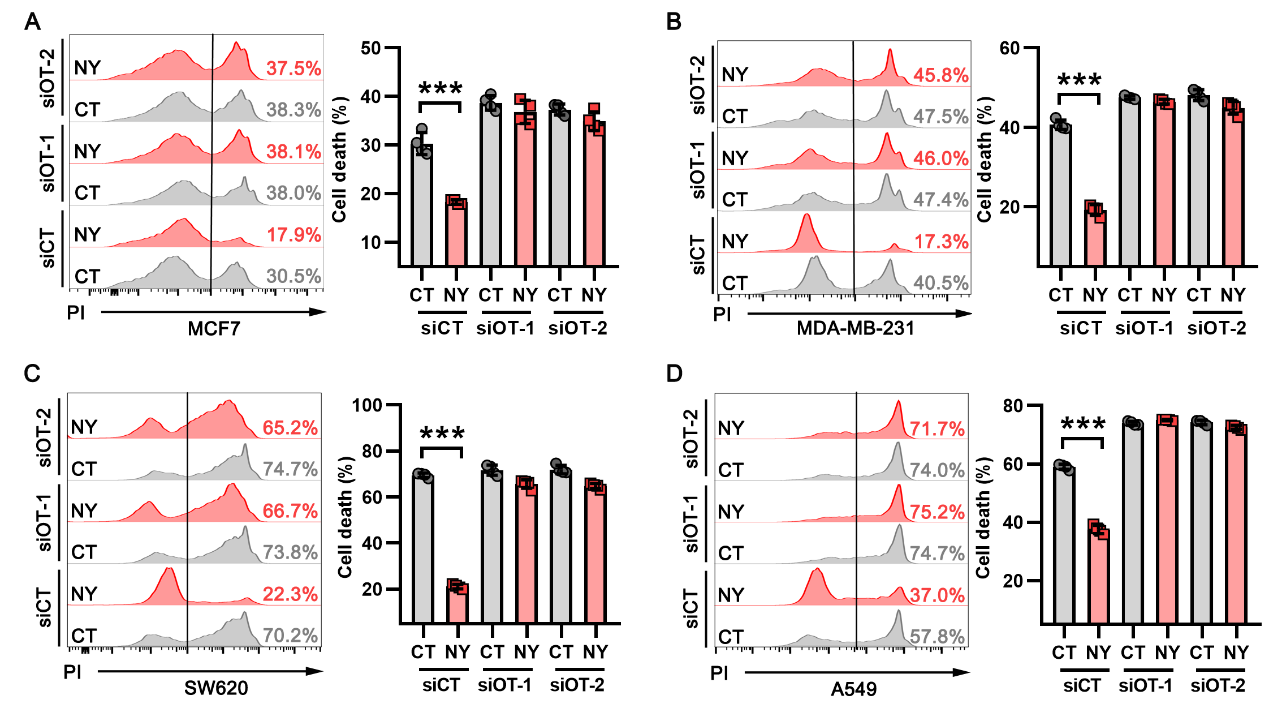


**Figure S10. Role of OTUB1 in NY-ESO-1-mediated anoikis resistance**

(A-D) Cell death analysis under suspension culture after OTUB1 knockdown in NY-ESO-1-expressing and control cells. Representative FACS graphs (left) and statistical analysis (right) of cell death in MCF-7 (A), MDA-MB-231 (B), SW620 (C), and A549 cells (D) are shown. Cells stably expressing control vector (CT) or NY-ESO-1 (NY) were transfected with OTUB1-targeting siRNAs (siOT-1, siOT-2), cultured in suspension for 72-120 h, and analyzed by PI staining. ****P*＜0.001.


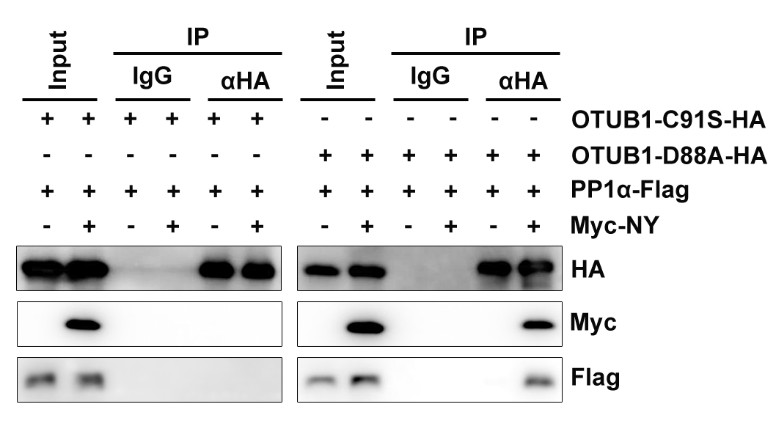


**Figure S11. Interactions between NY-ESO-1, PP1α, and OTUB1 mutants.**

Immunoblot analysis of the interactions between NY-ESO-1, PP1α, and OTUB1-C91S or OTUB1 D88A mutant in HEK293T cell lysates after IP with anti-Flag mAbs.

Table S1.

siRNA sequences

| siRNA | Target | Sense sequence (5'-3') | Reference |
| --- | --- | --- | --- |
| siP-1 | PP1α | AUGGAUUGAUUGUACAGAAAU | Ref[1] |
| siP-2 | PP1α | AAGAGACGCUACAACAUCAAA | Ref[1] |
| siUSP11-1 | USP11 | GCGCACAGCUGCAUGUCAUTT | Ref[2] |
| siUSP11-2 | USP11 | GGACCGUGAUGAUAUCUUCTT | Ref[2] |
| siUSP47 | USP47 | GACUCUGAUAGUGUAGCAUTT | Ref[3] |
| siUSP9X | USP9X | CUGUGAUUCAGCAACUCUATT | Ref[4] |
| siUSP10 | USP10 | CCACCUGAUGAAGUUCAUUTT | Ref[5] |
| siUSP5 | USP5 | GCCUCAAGCAGUUGGACAATT | Ref[6] |
| siOT-1 | OTUB1 | GACCAGGCCUGACGGCAACTT | Ref[7] |
| siOT-2 | OTUB1 | AGGAGUAUGCUGAAGAUGACA | This paper |
| siNY-1 | NY-ESO-1 | GCUUCAGGGCUGAAUGGAUTT | This paper |
| siNY-2 | NY-ESO-1 | CCGGCAACAUACUGACUAUTT | This paper |
| siCT | NA | UUCUCCGAACGUGUCACGUTT | This paper |

**Table S2.** Excel spreadsheet with the lists of proteins identified by LC-MS_MS

**Table S3.** Excel spreadsheet with the lists of genesets enriched in the NY-ESO-1 group

**Table S4.** Primer sequences used for qPCR

Primer sequences

| Primer | Sequence (5'-3') | Reference |
| --- | --- | --- |
| USP47-F | GGTCCCGAAAGAGATAGAAAATGC | Ref[3] |
| USP47-R | TTGGCCACATCTTCAAAGAGC |  |
| USP9X-F | AAGTGAAGCATGTCAGCGATT | Ref[4] |
| USP9X-R | GCCACACATAGCTCCACCA |  |
| USP10-F | GTGACACTTTGCCGAGAAC | Ref[5] |
| USP10-R | TCCGCCTCCACATTAGAAC |  |
| USP5-F | CCCTGCAGGCCAAGTCAGT | Ref[8] |
| USP5-R | CAGTCTAAGCCGAAGGTGAACTTC |  |

References

1. Davis RJ, Swanger J, Hughes BT, Clurman BE. The PP2A-B56 Phosphatase Opposes Cyclin E Autocatalytic Degradation via Site-Specific Dephosphorylation. Mol Cell Biol. 2017;37(8).

2. Ting X, Xia L, Yang J, He L, Si W, Shang Y, et al. USP11 acts as a histone deubiquitinase functioning in chromatin reorganization during DNA repair. Nucleic Acids Res. 2019;47(18):9721-40.

3. Ka HI, Lee S, Han S, Jeong AL, Park JY, Joo HJ, et al. Deubiquitinase USP47-stabilized splicing factor IK regulates the splicing of ATM pre-mRNA. Cell Death Discov. 2020;6:34.

4. Li X, Song N, Liu L, Liu X, Ding X, Song X, et al. USP9X regulates centrosome duplication and promotes breast carcinogenesis. Nat Commun. 2017;8:14866.

5. Chen Q, Hang Y, Zhang T, Tan L, Li S, Jin Y. USP10 promotes proliferation and migration and inhibits apoptosis of endometrial stromal cells in endometriosis through activating the Raf-1/MEK/ERK pathway. Am J Physiol Cell Physiol. 2018;315(6):C863-C72.

6. Li Y, Zhou J. USP5 Promotes Uterine Corpus Endometrial Carcinoma Cell Growth and Migration via mTOR/4EBP1 Activation. Cancer Manag Res. 2021;13:3913-24.

7. Sun XX, Challagundla KB, Dai MS. Positive regulation of p53 stability and activity by the deubiquitinating enzyme Otubain 1. EMBO J. 2012;31(3):576-92.

8. Kaistha BP, Krattenmacher A, Fredebohm J, Schmidt H, Behrens D, Widder M, et al. The deubiquitinating enzyme USP5 promotes pancreatic cancer via modulating cell cycle regulators. Oncotarget. 2017;8(39):66215-25.
