# Supplementary material for: NY-ESO-1 facilitates anoikis resistance and tumor metastasis by hijacking deubiquitinase OTUB1 to stabilize PP1α
Source: Cell Death Dis. 2025 Oct 6;16(1):682. doi: 10.1038/s41419-025-08017-w (PMC12500907; doi:10.1038/s41419-025-08017-w)

Original qPCR data and uncropped western blots for

**NY-ESO-1 facilitates anoikis resistance and tumor metastasis by hijacking deubiquitinase OTUB1 to stabilize PP1α**

Pengchao Zhang, *et al.*

*Corresponding author. Guizhong Zhang, gz.zhang@siat.ac.cn

Xiaolu Yang, [xyang@pennmedicine.upenn.edu](mailto:xyang@pennmedicine.upenn.edu)

Xiaochun Wan, xc.wan@siat.ac.cn

RT-qPCR raw data including *USP47*, *USP9X*, *USP10* and *USP5*

| **Sample** | **Target** | **Ct value (β-Actin)** | | | **Ct value (Target)** | | |
| --- | --- | --- | --- | --- | --- | --- | --- |
| **siCT** | ***USP47*** | 21.19 | 20.90 | 20.71 | 26.43 | 26.19 | 26.24 |
| **siUSP47** | ***USP47*** | 21.33 | 21.08 | 20.94 | 27.25 | 27.32 | 27.81 |
| **siCT** | ***USP9X*** | 21.19 | 20.90 | 20.71 | 26.36 | 26.50 | 26.50 |
| **siUSP9X** | ***USP9X*** | 21.25 | 21.30 | 21.05 | 28.71 | 28.66 | 28.67 |
| **siCT** | ***USP10*** | 21.19 | 20.90 | 20.71 | 26.26 | 26.32 | 26.15 |
| **siUSP10** | ***USP10*** | 21.18 | 20.76 | 20.67 | 28.14 | 28.02 | 28.15 |
| **siCT** | ***USP5*** | 21.19 | 20.90 | 20.71 | 28.74 | 28.68 | 28.89 |
| **siUSP5** | ***USP5*** | 21.07 | 20.70 | 20.70 | 31.33 | 31.36 | 31.45 |

Uncropped western blots
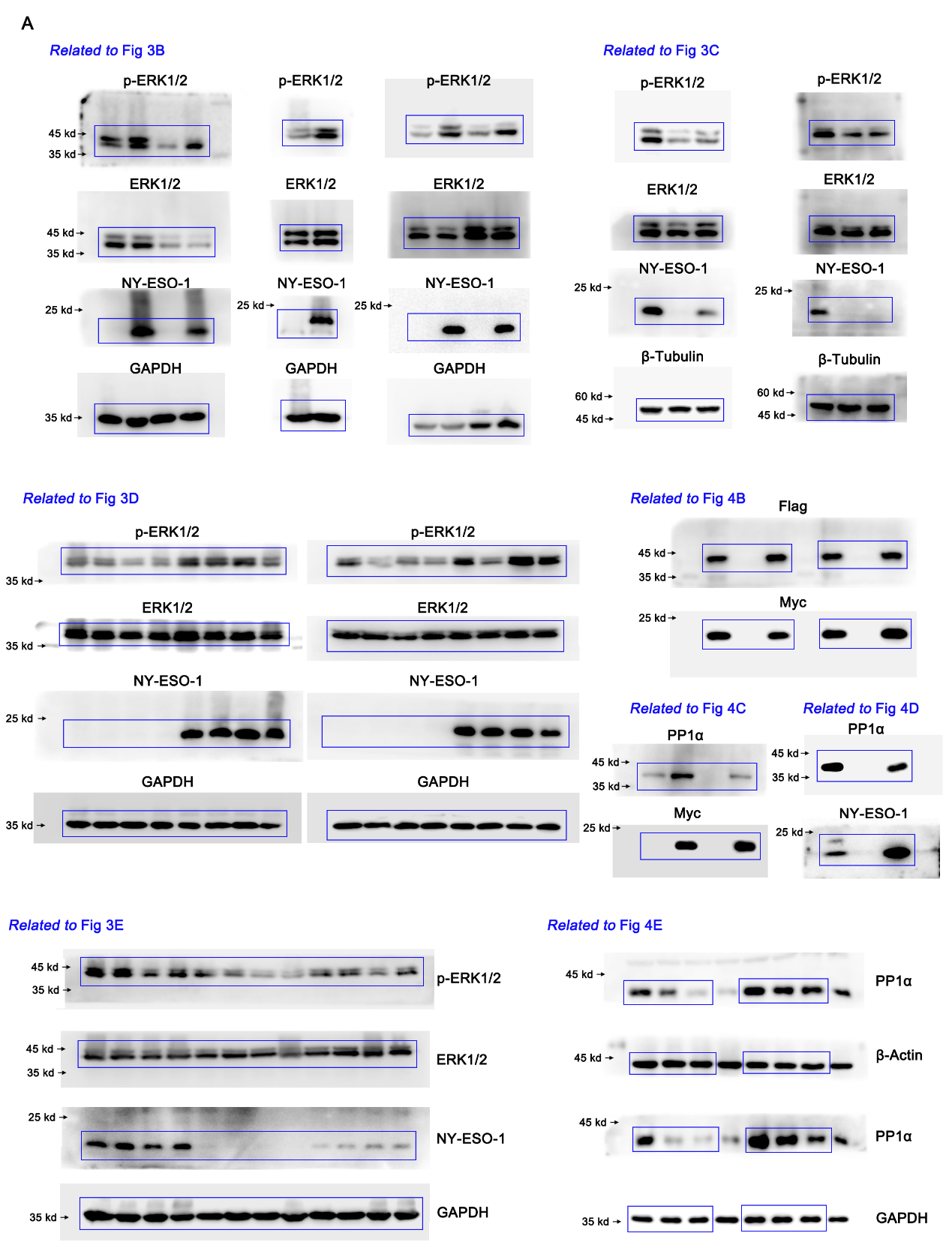

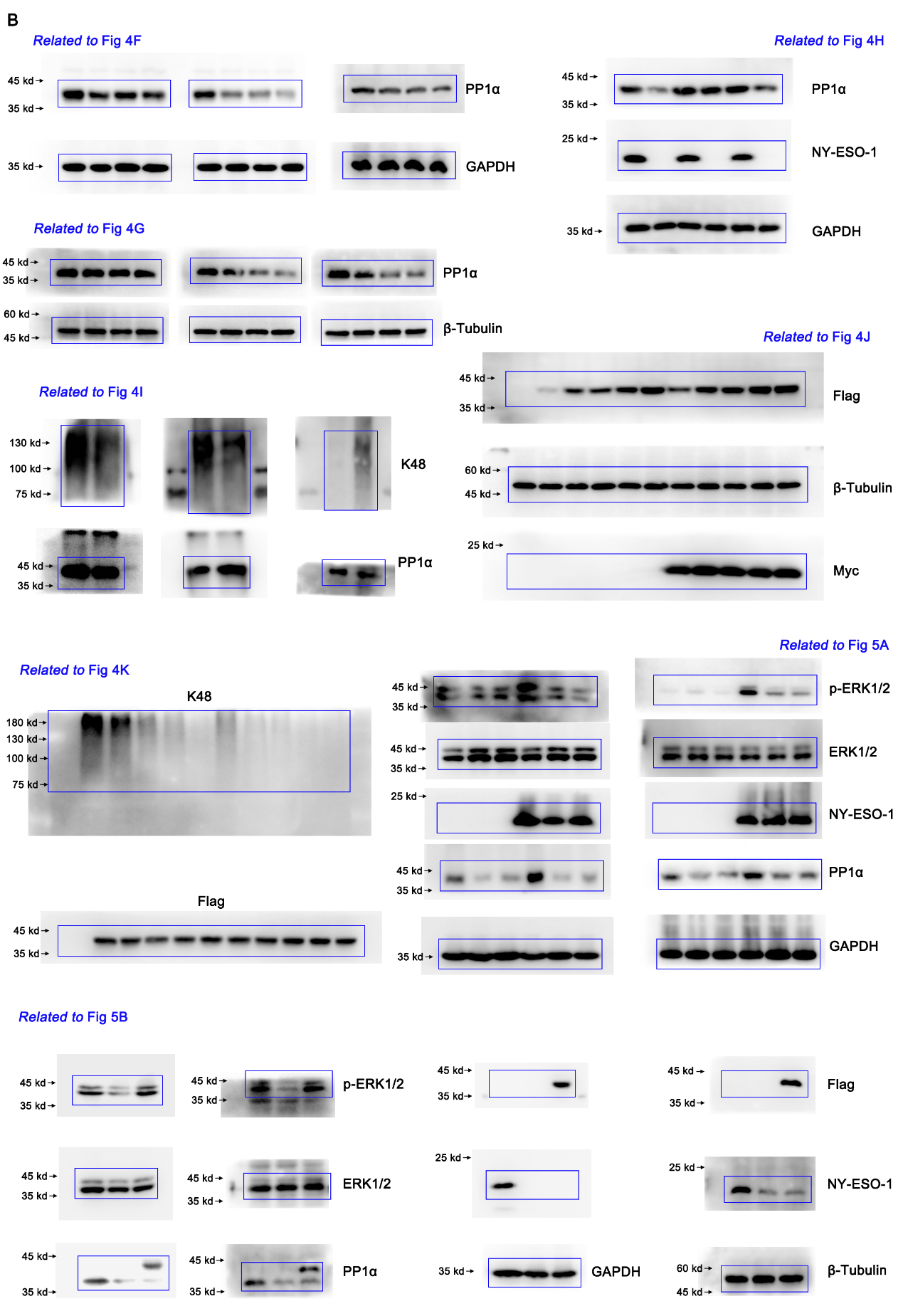

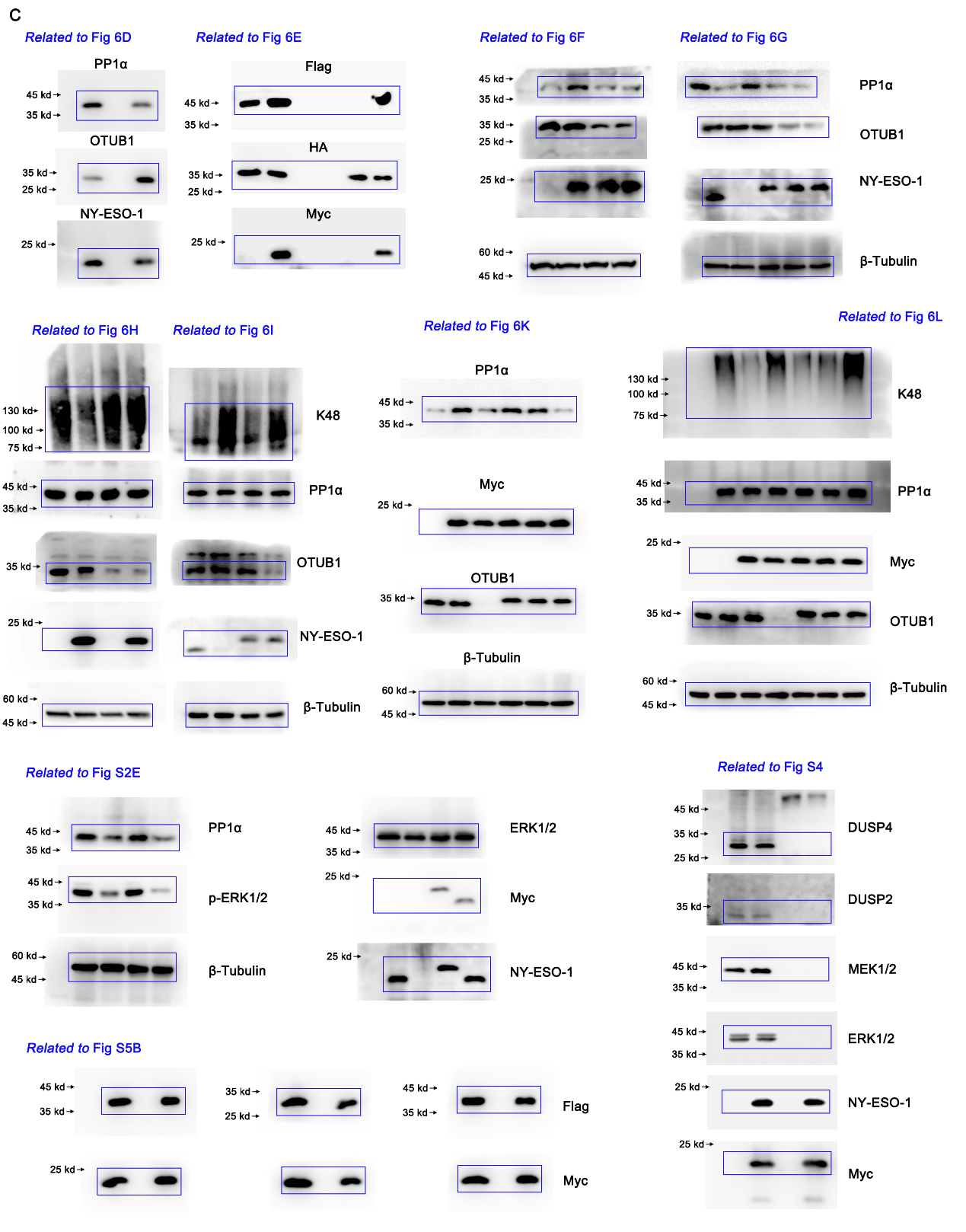

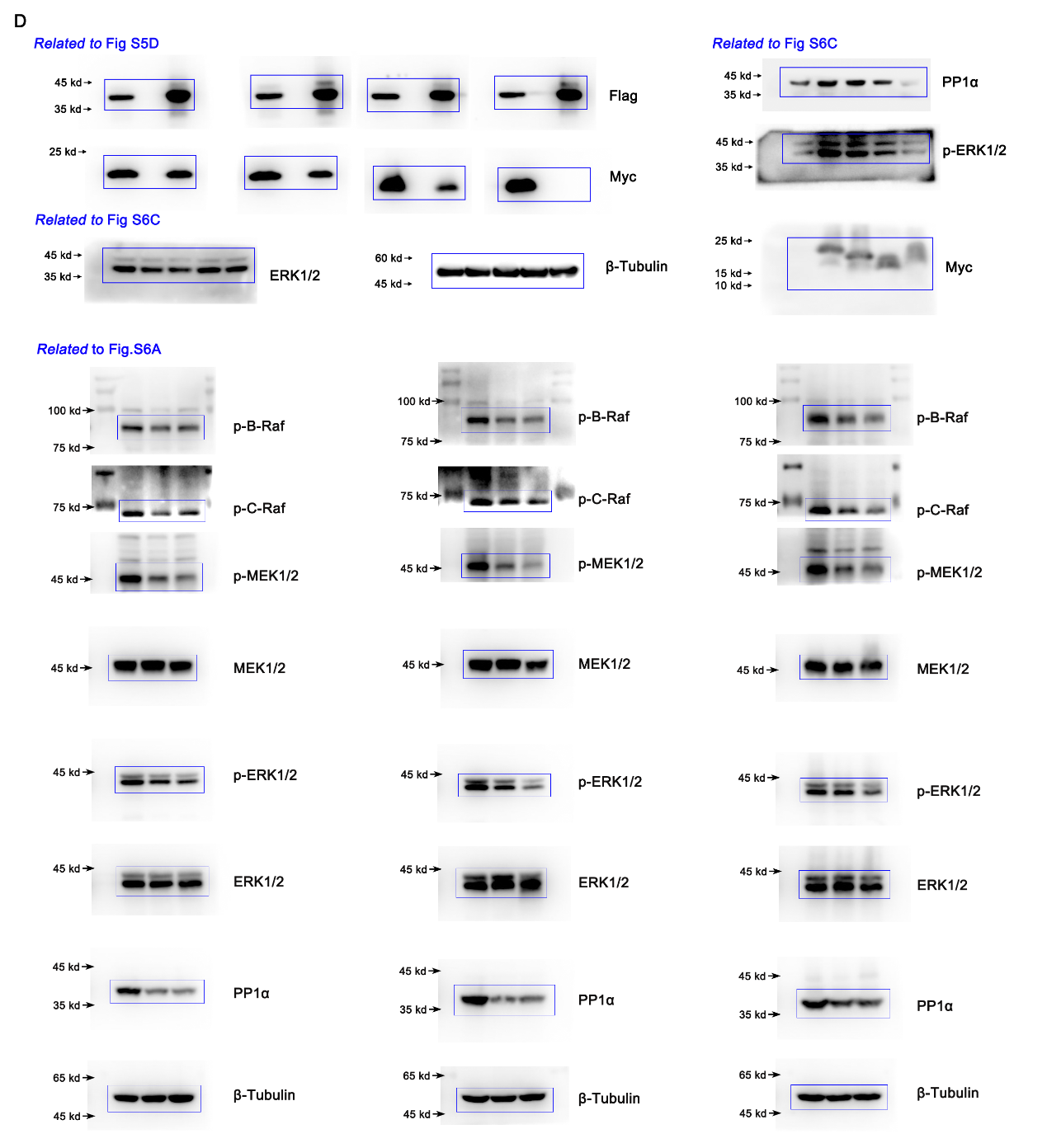


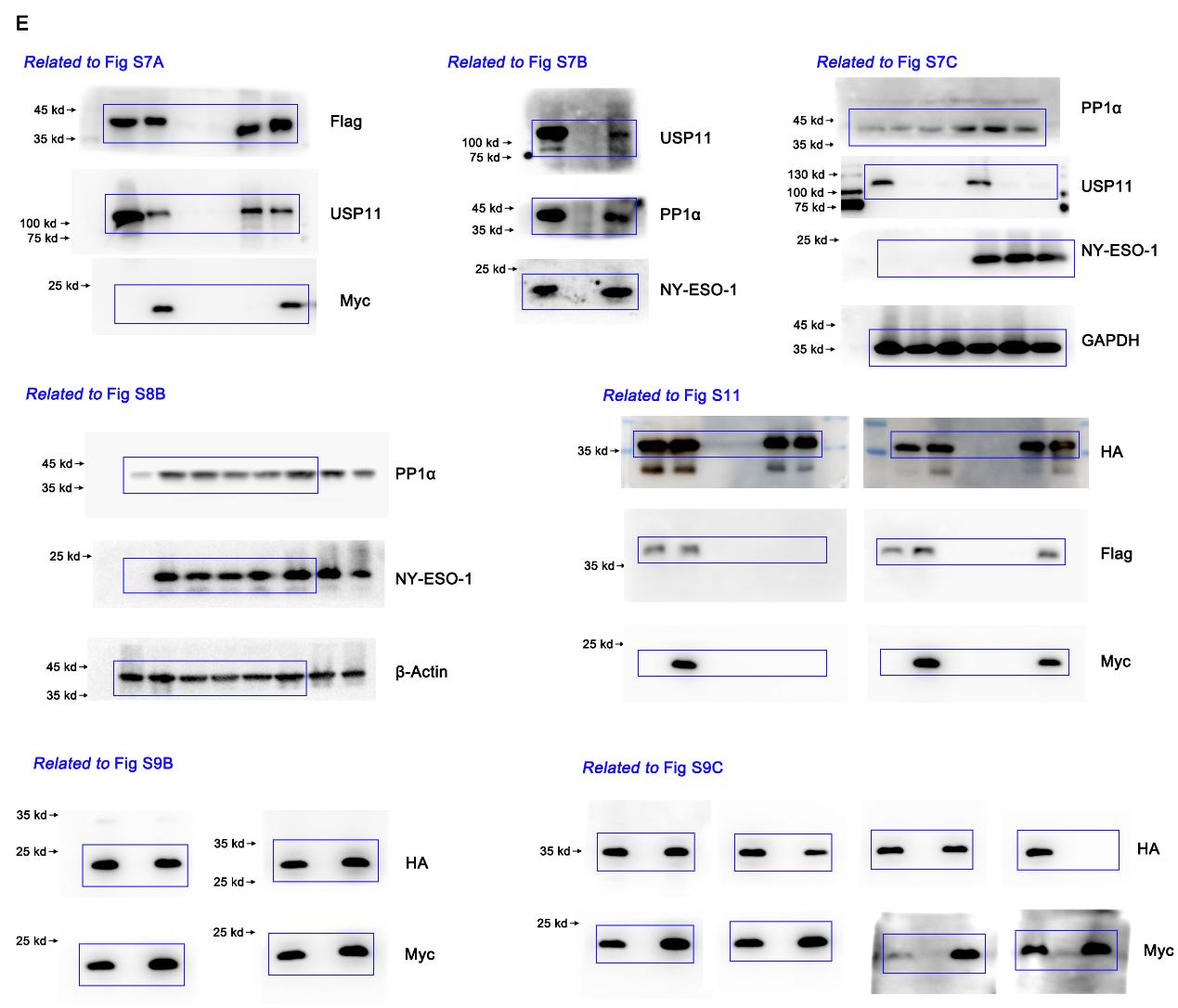

Supplement: Supplementary file 4 — Original data [file 41419_2025_8017_MOESM4_ESM.docx]
